# Association of germline variation with the survival of women with *BRCA1/2* pathogenic variants and breast cancer

**DOI:** 10.1038/s41523-020-00185-6

**Published:** 2020-09-10

**Authors:** Taru A. Muranen, Sofia Khan, Rainer Fagerholm, Kristiina Aittomäki, Julie M. Cunningham, Joe Dennis, Goska Leslie, Lesley McGuffog, Michael T. Parsons, Jacques Simard, Susan Slager, Penny Soucy, Douglas F. Easton, Marc Tischkowitz, Amanda B. Spurdle, kConFab Investigators, Rita K. Schmutzler, Barbara Wappenschmidt, Eric Hahnen, Maartje J. Hooning, HEBON Investigators, Christian F. Singer, Gabriel Wagner, Mads Thomassen, Inge Sokilde Pedersen, Susan M. Domchek, Katherine L. Nathanson, Conxi Lazaro, Caroline Maria Rossing, Irene L. Andrulis, Manuel R. Teixeira, Paul James, Judy Garber, Jeffrey N. Weitzel, SWE-BRCA Investigators, Anna Jakubowska, Drakoulis Yannoukakos, Esther M. John, Melissa C. Southey, Marjanka K. Schmidt, Antonis C. Antoniou, Georgia Chenevix-Trench, Carl Blomqvist, Heli Nevanlinna

**Affiliations:** 1 University of Helsinki, Department of Obstetrics and Gynecology, Helsinki University Hospital, Helsinki, Finland; 2 University of Turku and Åbo Akademi University, Turku Bioscience Centre, Turku, Finland; 3 University of Helsinki, Department of Clinical Genetics, Helsinki University Hospital, Helsinki, Finland; 4 Mayo Clinic, Department of Laboratory Medicine and Pathology, Rochester, MN USA; 5 University of Cambridge, Centre for Cancer Genetic Epidemiology, Department of Public Health and Primary Care, Cambridge, UK; 6 QIMR Berghofer Medical Research Institute, Department of Genetics and Computational Biology, Brisbane, QLD Australia; 7 CHU de Quebec Research Center, Genomics Center, Québec City, QC Canada; 8 Mayo Clinic, Department of Health Sciences Research, Rochester, MN USA; 9 University of Cambridge, Centre for Cancer Genetic Epidemiology, Department of Oncology, Cambridge, UK; 10 McGill University, Program in Cancer Genetics, Departments of Human Genetics and Oncology, Montréal, QC Canada; 11 University of Cambridge, Department of Medical Genetics, National Institute for Health Research Cambridge Biomedical Research Centre, Cambridge, UK; 12 Faculty of Medicine and University Hospital Cologne, University of Cologne, Center for Hereditary Breast and Ovarian Cancer, Cologne, Germany; 13 Faculty of Medicine and University Hospital Cologne, University of Cologne, Center for Molecular Medicine Cologne (CMMC), Cologne, Germany; 14 Erasmus MC Cancer Institute, Department of Medical Oncology, Family Cancer Clinic, Rotterdam, The Netherlands; 15 Medical University of Vienna, Dept of OB/GYN and Comprehensive Cancer Center, Vienna, Austria; 16 Odense University Hospital, Department of Clinical Genetics, Odence C, Denmark; 17 Aalborg University Hospital, Molecular Diagnostics, Aalborg, Denmark; 18 Aalborg University, Dept of Clinical Medicine, Aalborg, Denmark; 19 Perelman School of Medicine at the University of Pennsylvania, Department of Medicine, Abramson Cancer Center, Philadelphia, PA USA; 20 ICO-IDIBELL (Bellvitge Biomedical Research Institute, Catalan Institute of Oncology), CIBERONC, Molecular Diagnostic Unit, Hereditary Cancer Program, Barcelona, Spain; 21 Rigshospitalet, Copenhagen University Hospital, Center for Genomic Medicine, Copenhagen, Denmark; 22 Lunenfeld-Tanenbaum Research Institute of Mount Sinai Hospital, Fred A. Litwin Center for Cancer Genetics, Toronto, ON Canada; 23 University of Toronto, Department of Molecular Genetics, Toronto, ON Canada; 24 Portuguese Oncology Institute, Department of Genetics, Porto, Portugal; 25 University of Porto, Biomedical Sciences Institute (ICBAS), Porto, Portugal; 26 Peter MacCallum Cancer Center, Parkville Familial Cancer Centre, Melbourne, VIC Australia; 27 The University of Melbourne, Sir Peter MacCallum Department of Oncology, Melbourne, VIC Australia; 28 Dana-Farber Cancer Institute, Cancer Risk and Prevention Clinic, Boston, MA USA; 29 City of Hope, Clinical Cancer Genomics, Duarte, CA USA; 30 Pomeranian Medical University, Department of Genetics and Pathology, Szczecin, Poland; 31 Pomeranian Medical University, Independent Laboratory of Molecular Biology and Genetic Diagnostics, Szczecin, Poland; 32 National Centre for Scientific Research ‘Demokritos’, Molecular Diagnostics Laboratory, INRASTES, Athens, Greece; 33 Stanford Cancer Institute, Stanford University School of Medicine, Department of Medicine, Division of Oncology, Stanford, CA USA; 34 Monash University, Precision Medicine, School of Clinical Sciences at Monash Health, Clayton, VIC Australia; 35 The University of Melbourne, Department of Clinical Pathology, Melbourne, VIC Australia; 36 The Netherlands Cancer Institute-Antoni van Leeuwenhoek Hospital, Division of Molecular Pathology, Amsterdam, The Netherlands; 37 The Netherlands Cancer Institute-Antoni van Leeuwenhoek hospital, Division of Psychosocial Research and Epidemiology, Amsterdam, The Netherlands; 38 University of Helsinki, Department of Oncology, Helsinki University Hospital, Helsinki, Finland; 39 Örebro University Hospital, Department of Oncology, Örebro, Sweden

**Keywords:** Breast cancer, Cancer genetics, Genome-wide association studies

## Abstract

Germline genetic variation has been suggested to influence the survival of breast cancer patients independently of tumor pathology. We have studied survival associations of genetic variants in two etiologically unique groups of breast cancer patients, the carriers of germline pathogenic variants in *BRCA1* or *BRCA2* genes. We found that rs57025206 was significantly associated with the overall survival, predicting higher mortality of *BRCA1* carrier patients with estrogen receptor-negative breast cancer, with a hazard ratio 4.37 (95% confidence interval 3.03–6.30, *P* = 3.1 × 10^−9^). Multivariable analysis adjusted for tumor characteristics suggested that rs57025206 was an independent survival marker. In addition, our exploratory analyses suggest that the associations between genetic variants and breast cancer patient survival may depend on tumor biological subgroup and clinical patient characteristics.

## Introduction

Breast cancer is globally the leading cause of cancer-related mortality for women^1^. The average 5-year survival rate is 83–90% in the Western countries, substantially better than for many other cancers, but due to its high incidence, breast cancer still leads the statistics for cancer mortality in Europe and comes second in North America^1,2^. On an individual level, prognosis varies greatly, depending on both the inherent tumor biology and the stage of malignant progression at diagnosis. Women with early-stage, localized disease have a very good 5-year prognosis of ~97–99%, but for 10–15% of women, diagnosed with locally advanced disease, the expected 5- and 10-year survival rates range between 40–80% and 30–40%, respectively. Furthermore, the mortality associated with metastatic breast cancer is even higher, with median survival of <3 years^2–4^.

Currently, the prognosis of breast cancer patients is based on the tumor characteristics. Gene expression and copy number profiling^5–7^ or expression of marker proteins, like estrogen receptor (ER), progesterone receptor (PgR), human epidermal growth factor receptor 2, and marker of proliferation Ki-67, can be used to categorize breast cancers into biological subtypes with specific treatment recommendations and different estimates for patient survival^8,9^. Tumor grade is a histological measure of cellular growth pattern and the best individual prognostic factor^10^. The phase of tumor progression is approximated by clinical stage, which is based on the tumor size, as well as local and systemic spread of metastatic cells^11^. However, there is great variation in the survival rates between tumors with similar characteristics and stage. This variance has been suggested to have a heritable component, possibly consisting of genetic differences in metastatic potential and sensitivity to adjuvant therapy^12–18^. In addition, host factors, like tumor–microenvironment interaction, immune surveillance, and efficiency in drug metabolism may account for the genetic variability in breast cancer patient survival^19–21^.

Genetic determinants of patient prognosis and the treatment outcome prediction have been intensively sought using both candidate gene and genome-wide approaches, but only some of the discoveries have been successfully validated in subsequent studies. A meta-analysis of ten genome-wide association studies nominally validated 12 out of 45 earlier discoveries for survival from ER-positive or any breast cancer^22^. Most recently, the Breast Cancer Association Consortium (BCAC) reported two loci from chromosome 7, based on a well-powered meta-analysis of genome-wide association studies^23^.

In this study, we focused on the survival of women who carry germline pathogenic *BRCA1* or *BRCA2* variants. *BRCA1* and *BRCA2* are the two most important breast cancer susceptibility genes, with ~72 and 69% life-time risk of breast cancer and 44 and 17% risk of ovarian cancer, respectively^24^. *BRCA1/2*-deficient tumors have distinctive genomic aberration profiles, characterized by homologous recombination deficiency^25^, making them stand out as etiologically and phenotypically coherent groups of breast carcinomas. *BRCA1* risk variants are associated with high grade and triple-negative breast cancer, which both predict poor outcome. *BRCA2* risk variants predispose primarily to ER-positive breast cancer, but the risk of ER-negative breast cancer increases with age^26,27^. Meta-analyses on the survival of women with *BRCA1/2* variants and breast cancer have suggested no significant difference in comparison to noncarriers with phenotypically similar tumors^28–30^. However, a recent study suggested ER-positive breast cancer as an adverse indicator for cases with *BRCA2* variants, unlike for noncarriers^27^.

## Results

### Tumor characteristics

We investigated genetic survival associations in pathogenic variant carriers from the Consortium of Investigators of Modifiers of *BRCA1/2* (CIMBA), genotyped on the OncoArray^31,32^. Twenty-one independent studies participating in CIMBA had survival data available for germline carriers of pathogenic *BRCA1* variants (*n* = 3008) and 15 studies for carriers of *BRCA2* variants (*n* = 2,009; Supplementary Table 1). Primarily, we analyzed patient survival in relation to all-cause death, because this was most complete across the participating studies. As a sensitivity analysis, the discovered survival variants were also always assessed for breast cancer-specific death.

Data on tumor characteristics was available for about two thirds of patients (Table 1). The distribution of the tumor characteristics of *BRCA1* and *BRCA2* carriers agreed with previous reports, so that small, high-grade, and early-onset tumors had a relatively high frequency^26,33^. Tumors from *BRCA1* carriers were mostly ER-negative, while those from *BRCA2* carriers were largely ER-positive. Therefore, the main survival analyses were performed in parallel in the following patient groups: all *BRCA1* carriers, *BRCA1* carriers with ER-negative tumors, all *BRCA2* carriers, and *BRCA2* carriers with ER-positive tumors.

**Table 1:** Tumor characteristics of the patients included in the survival analysis.

Category	BRCA1 carriers *n*: 3008	(%)	(% Missing)	BRCA2 carriers *n*: 2009	(%)	(% Missing)
ER						
Negative	1510	(76.0%)		302	(22.1%)	
Positive	476	(24.0%)		1067	(77.9%)	
Not known	1022		(34.0%)	640		(31.9%)
PgR						
Negative	1409	(80.1%)		372	(32.9%)	
Positive	350	(19.9%)		759	(67.1%)	
Not known	1249		(41.5%)	878		(43.7%)
Grade						
1	40	(2.2%)		72	(5.8%)	
2	319	(17.6%)		526	(42.6%)	
3	1450	(80.2%)		636	(51.5%)	
Not known	1199		(39.9%)	775		(38.6%)
T						
≤2 cm	1054	(62.6%)		677	(59.0%)	
2–5 cm	576	(34.2%)		421	(36.7%)	
>5 cm	55	(3.3%)		50	(4.4%)	
Not known	1323		(44.0%)	861		(42.9%)
N						
Not affected	1305	(68.5%)		695	(53.4%)	
Affected	599	(31.5%)		606	(46.6%)	
Not known	1104		(36.7%)	708		(35.2%)
dg-age						
Mean (sd)	41.8 (9.3)			45 (9.6)		

The table summarizes the number of patients with specific recorded tumor characteristics, as well as the number of patients with no recorded data. The proportions are given in parenthesis, so that the categories with recorded data sum to 100%, whereas the proportion in category “not known” is reported in relation to the total of patients. dg-age was available for all patients. The last row of the table gives the average and standard deviation of the dg-age distribution.

*ER* estrogen receptor expression, *PgR* progesterone receptor expression, *T* tumor size category, *N* status of axillary lymph nodes, *dg-age* diagnosis age, *sd* standard deviation.

### rs57025206 predicts survival of *BRCA1* carriers with ER-negative breast cancer

**Fig. 1:**
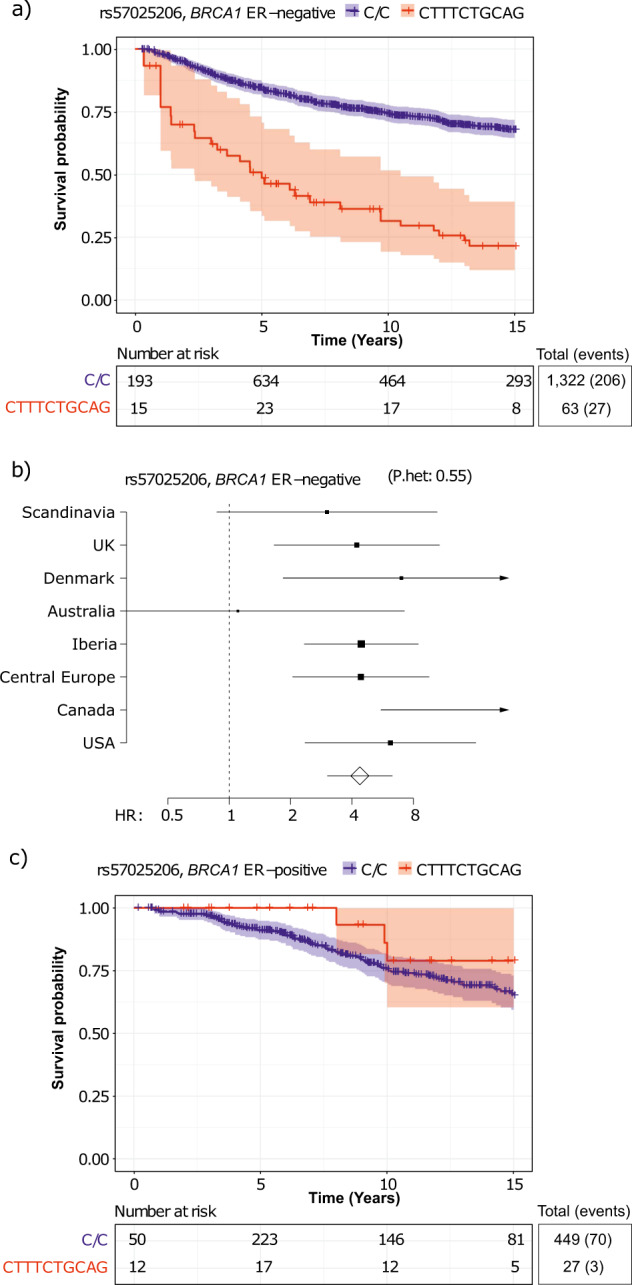
Survival variant rs57025206. **a** Kaplan–Meier plot on the survival of ER-negative breast cancer patients carrying pathogenic *BRCA1* variants, stratified by rs57025206. **b** Forest plot of HR associated with rs57025206 across country groups. (P.het: *P*-value against between-study heterogeneity). **c** Kaplan–Meier plot on the survival of ER-positive breast cancer patients carrying pathogenic *BRCA1* variants, stratified by rs57025206.

We analyzed the association of germline genetic variants with the overall survival after the first primary breast cancer diagnosis in carriers of pathogenic *BRCA1* or *BRCA2* variants, using Cox regression. The analyses were adjusted for age at breast cancer diagnosis and stratified by country group to account for underlying genetic and clinical differences between populations. A single variant, rs57025206 (minor allele frequency in European population, MAF_EUR_, 0.027; info score 0.97 for imputed variant), a nine base pair insertion in an intergenic region of 3p21.2, was identified as a highly significant survival marker for ER-negative breast cancer patients carrying *BRCA1* pathogenic variants, with a hazard ratio (HR) of 4.37 (95% confidence interval (CI) 3.03–6.30, *P* = 3.1 × 10^−9^, Fig. 1). Furthermore, a multivariable analysis adjusted for tumor grade, size, PgR expression status, and lymph node involvement, suggested that the association is independent of these tumor characteristics, with rs57025206-associated HR: 6.19, 95% CI: 3.73–10.3. A similar trend was observed in the analysis of breast cancer-specific death, although the number of patients with available data was much smaller (Supplementary Table 2).

The survival association was specific to women with ER-negative breast cancer, since in the analysis of all *BRCA1* carriers the HR was attenuated (HR: 2.12, 95% CI: 1.55–2.91, Supplementary Fig. 1) and not seen in those with ER-positive breast cancer (*n*: 476, HR: 0.52, 95% CI: 0.17–1.60, Fig. 1c). In the analysis of *BRCA2* carriers, rs57025206 was not associated with patient survival (HR: 0.94, 95% CI: 0.59–1.51).

### The main analyses suggested nine further survival loci

We used a looser discovery threshold *P*-value, *P* < 5 × 10^−7^, than in the traditional genome-wide setting to characterize loci that potentially modify the patient survival and may form a basis for future research hypotheses. In the analysis of the overall survival of *BRCA1* carriers, six variants exceeded the selected threshold (Table 2 and Supplementary Fig. 2). The risk magnitude was proportionally associated with the number of risk alleles for two variants, rs59010985 and rs537497819, whereas the other four variants followed a dominant inheritance pattern. The analysis of *BRCA1* carriers with ER-negative breast cancer identified four further potential variants influencing survival (Table 2 includes also rs57025206, presented above). No variant exceeded the significance threshold in the analysis of *BRCA2* carriers, alone. However, a meta-analysis of *BRCA1* and *BRCA2* carriers (see below) suggested germline variants associated with survival in both of these groups.

**Table 2:** Variants associated with the survival of breast cancer patients carrying pathogenic germline variants in *BRCA1*.

Variant	Chr:position	Effect allele	Reference allele	EAF	Analysis group	Genetic model	HR [95% CI]	P.LR	BFDP (1.3)	BFDP (1.8)	BFDP (2.5)	Proportional hazards assumption
rs12126206	1:245642358	A	T	0.076	BRCA1 all	Dominant	1.86 [1.50–2.32]	1.7E−07	0.58	0.03	0.01	0.95
rs5028286	8:14831991	G	A	0.10	BRCA1 all	Dominant	1.74 [1.42–2.12]	3.3E−07	0.62	0.06	0.04	0.84
rs736418	9:136359182	G	A	0.23	BRCA1 all	Dominant	0.60 [0.49–0.73]	2.4E−07	0.88	0.33	0.26	0.49
rs147857072	14:36719644	C	T	0.010	BRCA1 all	Dominant	3.41 [2.32–5.00]	4.0E−07	0.96	0.02	0.00	0.45
rs59010985	17:9945581	T	C	0.16	BRCA1 all	Per-allele linear	0.58 [0.47–0.72]	1.3E−07	0.92	0.39	0.30	0.17
rs537497819	18:24664422	GT	G	0.59	BRCA1 all	Per-allele linear	1.49 [1.27–1.74]	4.3E−07	0.71	0.30	0.29	0.0034^a^
rs1829314	2:123530634	T	C	0.027	BRCA1 ER-	Dominant	0.00 [0.00–0.00]	1.2E−07	–	–	0.00	0.99
rs149851278	3:1905589	G	T	0.13	BRCA1 ER-	Per-allele linear	0.43 [0.29–0.63]	4.3E−07	1.00	0.97	0.92	0.09
rs57025206	3:51788456	CTTTCTGCAG	C	0.023	BRCA1 ER-	Per-allele linear	4.37 [3.03–6.30]	3.1E−09	0.23	0.00	0.00	0.75
rs11245414	10:126582635	T	A	0.81	BRCA1 ER-	Per-allele linear	0.56 [0.46–0.70]	2.7E−07	0.83	0.17	0.11	0.16
rs78150318	14:67931241	T	C	0.029	BRCA1 ER-	Dominant	0.00 [0.00–0.00]	1.3E−07	–	–	0.00	0.83

The “Proportional hazards assumption” column gives *P*-values for the assumption that the variant effect remains constant during the follow-up time.

*Chr* chromosome, *EAF* effect allele frequency in data, *HR* hazard ratio, *CI* confidence interval, *P.LR* *P*-value from likelihood-ratio test, *BFDP* Bayesian false discovery probability.

aThe hazard associated with the SNP was 1.89 [1.47–2.42] during the first 5 years after the diagnosis and 1.24 [1.01–1.53] later.

The reliability of the findings was estimated with a Bayesian false discovery probability (BFDP) and by assessing the between-strata (country group) heterogeneity. Ten out of the 11 *BRCA1*-associated survival variants had BFDP ≤ 0.33 for at least one of the three tested prior maximum effect sizes (Table 2). For these ten variants, there was little heterogeneity between countries (Supplementary Fig. 3, *P* against heterogeneity >0.1 for all variants). Moreover, the variant effect sizes were consistent in multivariable models adjusted for tumor pathologic characteristics and in the analyses of breast cancer-associated death (Supplementary Table 2), suggesting a consistent effect throughout the data.

Most of the identified survival variants complied with the proportional hazards assumption, suggesting a constant HR over time, but rs537497819 was especially associated with poor short-term prognosis, the effect leveling out after the first 5 years following the diagnosis (Table 2).

### Meta-analysis suggested four variants with the consistent survival effect in *BRCA1* and *BRCA2* carriers

The correlation between variant effect sizes in the *BRCA1* and *BRCA2* survival analyses was quite low (Pearson’s *R* = 0.0065 [95% CI 0.0059–0.0072]), suggesting no overall trend for similar genetic survival effects in these patient groups. Nevertheless, a fixed-effects meta-analysis highlighted four candidate variants, which had consistent effect on patient survival in both groups (Table 3, Supplementary Table 3 and Supplementary Fig. 4).

**Table 3:** Variants associated with the survival of breast cancer patients in the meta-analysis of *BRCA1* and *BRCA2* carriers.

Variant	Chr:position	Effect allele	Reference allele	EAF	Analysis group	Genetic model	BRCA1 HR [95% CI]	BRCA2 HR [95% CI]	Meta-analysis HR [95% CI]	P.LR	BFDP (1.3)	BFDP (1.8)	BFDP (2.5)
rs551383190	1:91487202	C	A	0.14	BRCA1/BRCA2	Per-allele linear	0.57 [0.45–0.74]	0.66 [0.50–0.88]	0.61 [0.51–0.73]	7.0E−08	0.46	0.05	0.04
rs117422049	2:122474495	A	G	0.011	BRCA1/BRCA2	Per-allele linear	2.67 [1.74–4.10]	3.05 [1.80–5.17]	2.80 [1.89–4.13]	2.6E−07	0.99	0.64	0.25
rs4879914	9:35621336	C	T	0.55	BRCA1/BRCA2	Per-allele linear	1.26 [1.11–1.43]	1.36 [1.16–1.59]	1.30 [1.17–1.44]	4.7E−07	0.31	0.21	0.25
rs2320070	14:22380540	T	C	0.74	BRCA1/BRCA2	Per-allele linear	1.42 [1.19–1.68]	1.35 [1.10–1.65]	1.39 [1.22–1.58]	4.6E−07	0.44	0.20	0.22

*Chr* chromosome, *EAF* effect allele frequency in data, *HR* hazard ratio, *CI* confidence interval, *P.LR* *P*-value from likelihood-ratio test, *BFDP* Bayesian false discovery probability.

### Four variants may have age-dependent survival effects for *BRCA2* carriers

The lack of positive findings in the *BRCA2*-specific analyses prompted us to consider the possibility that there may be confounding factors, so that the genetic survival associations would depend on tumor and patient characteristics. We had tumor pathology data available only for a subgroup of the study subjects, and lacked statistical power to investigate the interaction between genetic variants and tumor characteristics. However, the age at diagnosis was available for all cases, and to investigate potential age-dependent genetic survival effects, we included in the Cox regression model a covariate coded as the product of the variant genotype and diagnosis age (continuous). The model containing the interaction term was tested against a nested model without interaction. Likelihood-ratio test *P*-values (Table 4) were corrected for multiple testing using the Benjamini–Hochberg method and variants with false discovery rate (FDR) ≤ 0.33 were considered as potential discoveries. Four variants, all from the analysis of *BRCA2* carriers, passed the threshold. For illustrative purposes, the HRs in Table 4 are presented separately for two age groups, even though the regression model suggested that the interaction between the variant survival effect and the diagnosis age is continuous (Supplementary Fig. 5). The variant effect sizes were consistent in multivariable models, including tumor pathologic characteristics, as well as in the analysis of breast cancer-specific death (Supplementary Table 4).

**Table 4:** Variants, whose association on patient survival was dependent on the age at the breast cancer diagnosis.

Variant	Chr:position	Effect allele	Reference allele	EAF	Analysis group	Genetic model	HR [95% CI] (under 40 years)	HR [95% CI] (over 40 years)	P.LR	FDR	Proportional hazards assumption
rs372812554	3:34507804	TTTA	T	0.26	BRCA2 all	Per-allele interaction with age	1.75 [1.33–2.31]	0.75 [0.59–0.96]	2.7E−07	0.33	0.71
rs2109815	7:28393925	A	G	0.27	BRCA2 all	Per-allele interaction with age	1.65 [1.24–2.19]	0.75 [0.59–0.95]	2.9E−07	0.33	0.14
rs35431863	8:62520183	G	GA	0.49	BRCA2 all	Per-allele interaction with age	0.55 [0.43–0.71]	1.32 [1.09–1.60]	8.2E−08	0.33	0.58
rs11255420	10:7915592	T	C	0.24	BRCA2 all	Per-allele interaction with age	0.59 [0.41–0.83]	1.55 [1.24–1.93]	1.2E−07	0.33	0.40

The “Proportional hazards assumption” gives *P*-values for the assumption that the survival model described in the regression model containing the interaction term remains constant during the follow-up time.

*Chr* chromosome, *EAF* effect allele frequency in data, *HR* hazard ratio, *CI* confidence interval, *P.LR* *P*-value from likelihood-ratio test, *FDR* false discovery rate.

ER-negative breast cancer is more common for postmenopausal than premenopausal *BRCA2* carriers^26,33^. To test whether the age-dependent survival association of the four variants (Table 4), was a hidden association with the tumor subtype, we analyzed the survival separately in age- and subtype-stratified subgroups, including only patients with data on ER expression available (Table 1). This analysis suggested that the ER status did not explain the age-dependent survival association. For two variants (rs2109815 and rs35431863), the age-dependent survival association was similar in ER-positive and ER-negative patient groups. For the other two variants (rs372812554 and rs11255420), the survival association did not vary by age of diagnosis in the ER-negative patient group, and the group resembled the younger ER-positive patient group (Supplementary Table 5).

### The positional and eQTL analyses reveal potential target genes

None of the variants we found associated with survival was located on a protein-coding region. Therefore, we took two parallel approaches, positional and gene expression based, to identify potential target genes. A target gene was considered to have a strong positional evidence, if the survival variant or any regulatory variant in linkage disequilibrium was located on an experimentally validated enhancer of the target gene in a relevant tissue (mammary, ovarian, blood, and adipose)^34^, based on the 1000 genomes and Encode data^35,36^. This is indicated in the Table 5, column “Positional target tissue”. If no other evidence was available, the target gene was assumed the closest gene (no “Positional target tissue” mentioned in Table 5). For the gene expression-based approach, we used eQTL data from public databases covering various human tissues and cell types (Supplementary Data 1 and 2).

**Table 5:** Target genes.

Survival SNP	Effect allele	HR HR_young_/HR_old_	eQTL SNP	*D*′	*R*^2^	Correlated eQTL allele	Effect on target gene	eQTL target gene	eQTL target tissue	Positional target gene	Positional target tissue
rs12126206	A	1.86								KIF26B	
rs5028286	G	1.74								SGCZ	
rs5028286										MIR383	
rs736418	G	0.60	rs11535669	0.93	0.06	G	Low expression	CACFD1	Adipose	CACFD1	Breast, ovary, and blood
rs736418			rs11535669	0.93	0.06	G	High expression	MED22	Blood	MED22	Ovary and blood
rs736418			rs76643124	1.00	0.01	G	High expression	RALGDS	Breast		
rs736418			rs76643124	1.00	0.01	G	Low expression	ABO	Adipose and blood		
rs736418										SLC2A6	Breast, ovary, and blood
rs147857072	C	3.41								MBIP	
rs59010985	T	0.58	rs11656239	1.00	0.97	T	High expression^a^	GAS7	Blood	GAS7	Blood
rs537497819	GT	1.49								CHST9	
rs57025206	INS	4.37								MANF	Breast, ovary, and blood
rs57025206										DCAF1	Breast, ovary, and blood
rs57025206										RBM15B	Breast, ovary, and blood
rs57025206										RAD54L2	Breast, ovary, and blood
rs57025206										TEX264	Breast, ovary, and blood
rs57025206			rs1476290	1.00	0.92	A	High expression	MIR135A1	Adipose		
rs57025206			rs72945708	0.95	0.66	G	Low expression	GRM2	Adipose	GRM2	Breast, ovary, and blood
rs11245414	T	0.56	rs4478950	0.80	0.48	T	High expression^a^	ZRANB1	Blood	ZRANB1	Breast, ovary, and blood
rs11245414										CTBP2	Breast, ovary, and blood
rs1829314	T	0.00									
rs78150318	T	0.00								TMEM229B	
rs78150318										RAD51B	
rs372812554	INS	1.75/0.75								LINC01811	
rs2109815	A	1.65/0.75								CREB5	Blood
rs35431863	G	0.55/1.32								ASPH	Breast, ovary, adipose, and blood
rs11255420	T	0.59/1.55								GATA3	Breast and blood
rs11255420			rs9633771	0.97	0.85	A	High expression	ATP5C1	Lung		
rs551383190	C	0.61								ZNF644	Breast, ovary, and blood
rs117422049	A	2.8	rs13000440	0.88	0.10	T	High expression^a^	CLASP1	Blood	CLASP1	Breast, ovary, and blood
rs117422049			rs12616209	1.00	0.14	T	High expression	NIFK	Blood	NIFK	Breast, ovary, and blood
rs117422049										NIFK-AS1	Breast, ovary, and blood
rs117422049			rs6541775	0.88	0.07	T	Low expression	TFCP2L1	Blood		
rs4879914	C	1.3	rs4879914	1.00	1.00	C	High expression	DNAJB5	Breast		
rs4879914			rs1538537	0.83	0.27	T	High expression	ARHGEF39	Breast	ARHGEF39	Breast, ovary, and blood
rs4879914			rs4879915	1.00	0.97	C	Low expression	TPM2	Blood	TPM2	Breast, ovary, and blood
rs4879914			rs4879915	1.00	0.97	C	Low expression	GBA2	Blood	GBA2	Breast, ovary, and blood
rs4879914			rs2297876	0.93	0.28	G	High expression	RUSC2	Blood	RUSC2	Breast, ovary, and blood
rs4879914			rs2297876	0.93	0.28	G	High expression^a^	CD72	Blood	CD72	Breast, ovary, and blood
rs4879914			rs2071675	0.86	0.04	A	Low expression^a^	TMEM8B	Blood	TMEM8B	Breast, ovary, and blood
rs2320070	T	1.39	rs28501628	0.98	0.21	A	Low expression	TRAV19	Blood	TRAV	

^a^The same or linked SNP was an eQTL to the same gene with opposite direction in other tissues than blood.

**Fig. 2:**
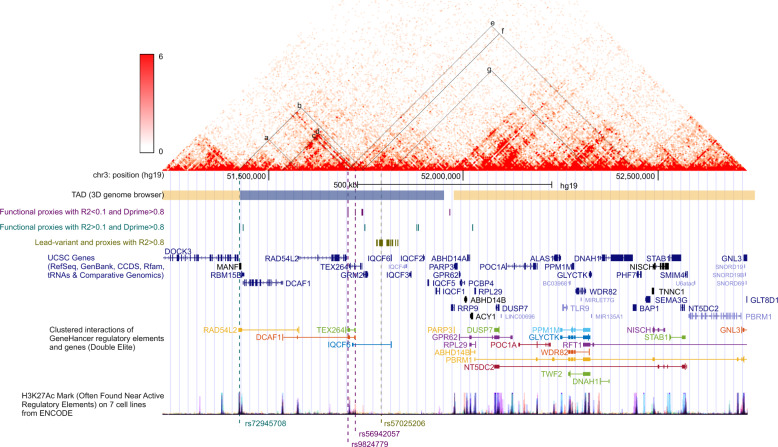
Potential target genes on a survival locus 3p21. The heatmap visualizes the strength of association between two genomic loci in chromosome conformation capture data from human mammary epithelial cells. The graph has been created and the topologically associated domains (TAD) estimated with 3D genome browser, promoter.bx.psu.edu/hi-c/. The UCSC browser tracks visualize genomic annotation for the region. The three custom tracks indicate the positions of variants in linkage disequilibrium with rs57025206. The vertical dashed lines indicate the positions of the lead variant rs57025206 and three linked regulatory variants. UCSC genes track visualizes the positions of exons and introns for all genes on the region. The GeneHancer track displays the interactions of distant enhancers and gene transcription start sites (TSSs). The histone mark track shows the positions of H3K27Ac from the Encode project. In the heatmap, seven positions (**a**–**e**) have been marked in the heatmap to indicate the probability of physical interaction between variants linked with rs57025206 and their target genes based on positional and eQTL analysis (Supplementary Data 1 and 2) as follows: (a) *RAD54L2* TSS and rs72945708 on enhancer GH03J051383 targeting *RAD54L2*. (b) *GRM2* TSS and *GRM2* eQTL variant rs72945708 on enhancer GH03J051383. (c) *DCAF1* TSS and rs9824779 on enhancer GH03J051668 targeting *DCAF1*. (d) *DCAF1* TSS and rs56942057 on enhancer GH03J051684 targeting *DCAF1*. (e) *BAP1* TSS and *BAP1* eQTL variant rs9824779 on enhancer GH03J051668. (f) *BAP1* TSS and *BAP1* eQTL variant rs60497133. (g) *MIR135A* TSS and *MIR135A* eQTL variants rs1476290 and rs1605067.

rs57025206 and its proxy variants in high linkage disequilibrium (*R*^2^ > 0.8 in the European population, green track named “lead variant and proxies with *R*^2^ > 0.8” in Fig. 2) are co-located with a cluster of *IQCF*-genes (*IQCF1–6*), expressed exclusively in the testis. These variants have eQTL associations with altogether five genes in different tissues, for example, with *MIR135A* in adipose tissue. However, the region does not contain annotated regulatory elements. Furthermore, it is not in physical contact with any transcription start site (TSS) in human mammary epithelial cells (HMEC), suggesting that this region does not contain any HMEC-specific enhancer (see, for example, position “g” in the heatmap of Fig. 2). Furthermore, the MAFs of these variants range from 2.2 to 3.2% in the European population, but between 12 and 27% globally, questioning whether the causal variant would be rs57025206 or any of the proxies with *R*^2^ > 0.8.

The rs57025206 haploblock (with *D*′ > 0.8) covers a larger genomic region with functional variants located on enhancers regulating several protein-coding genes (turquoise and green tracks in Fig. 2, Table 5 and Supplementary Data 1). For example, rs56942057 (MAF_EUR_ 0.1%, MAF_GLOBAL_ 4.6%, *D*′_EUR_ 1.0, and *D*′_GLOBAL_ 0.34) and rs9824779 (MAF_EUR_ 0.1%, MAF_GLOBAL_ 3.9%, *D*′_EUR_ 1.0, and *D*′_GLOBAL_ 0.32) are located in enhancers of *DCAF1*, GH03J051684, and GH03J051668, respectively. Both variants affect conserved sequences of transcription factor binding motifs. Furthermore, the variant positions and *DCAF1* TSS are in physical contact in HMEC (positions “c” and “d” in Fig. 2). These two (rs56942057 and rs9824779) and rs60497133 were eQTL variants for *BAP1* in thyroid (Supplementary Data 2), but in HMEC there was no contact between the variant positions and *BAP1* TSS (Fig. 2, positions “e” and “f”), suggesting that the regulatory associations of these variants and *BAP1* may not exists in mammary epithelial cells. A third potentially causal variant, rs72945708 (MAF_EUR_ 2.0%, MAF_GLOBAL_ 18%, *D*′_EUR_ 0.95, and *D*′_GLOBAL_ 0.71), is located on a *RAD54L2* enhancer GH03J051383 and associated with the expression of *GRM2* in adipose tissue. However, this variant does not change conserved sequence of any known transcription factor binding motif, and the topological association between the variant locus and *RAD54L2* or *GRM2* TSS cannot be seen in HMEC (positions “a” and “b” in Fig. 2, respectively). Thus, the plausible culprit gene of the rs57025206 locus is *DCAF1*, an E3 ubiquitin ligase substrate receptor targeting, e.g., TP53, ER-alpha, and EZH2.

We performed a similar target gene analysis for the 17 further survival candidate variants (Tables 2–4) using positional and eQTL data (Table 5 and Supplementary Data 1 and 2). For only one variant, rs59010985, there was a single clear target gene, *GAS7*, which was supported by both analyses. The majority of the other variants could be divided into three categories. Some variants had multiple potential target genes. For example, variants in linkage disequilibrium with rs4879914, detected in the *BRCA1*–*BRCA2* meta-analysis, were eQTLs for several genes in white blood cells. Positional evidence supported the same array of target genes, in both mammary epithelial cells and white blood cells. For some other loci, like *ASPH* and *CREB5*, the target gene prediction was based merely on the regulatory variants located on active enhancers in relevant tissues, with no eQTL evidence. Furthermore, for six loci, the target gene was assumed the closest gene. One of the 17 survival variants was located in an intergenic region with no apparent target gene.

**Fig. 3:**
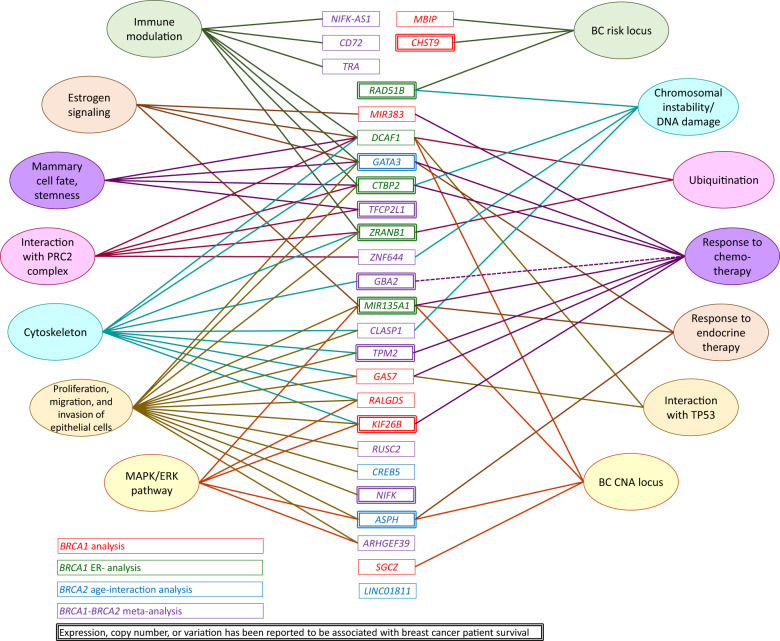
Functional clustering of the target genes based on published literature. For details and references, see Supplementary Table 6. PRC2 polycomb repressor complex 2, BC breast cancer, CNA copy number aberration.

To detect common pathways or recurrent cellular or molecular functions, we performed a systematic literature review (Fig. 3 and Supplementary Table 6). Relevant literature was available for 26 out of the 40 target genes listed in Table 5, when searched from PubMed with the keyword combinations described in the “Methods” section. Twelve of the genes had previously been connected with breast cancer patient survival, based on either mRNA or protein expression, germline genetic variation, or copy number change in mammary tumors (Fig. 3 and Supplementary Table 6). Fourteen of the target genes were associated with proliferative and migratory capacity of mammary or other epithelial cells. This association was mediated via two primary mechanisms: regulation of MAPK/ERK pathway activity or modulation of cytoskeletal proteins. Target genes in two out of the four loci associated with the survival of *BRCA1* carriers with ER-negative breast cancer (*DCAF1*, *ZRANB1*, and *CTBP2*) and two genes from the *BRCA1*–*BRCA2* meta-analysis (*ZNF644* and *TFCP2L1*) affect the regulation of chromatin state either directly or via polycomb repressor complex 2 (PCR2). Eight target genes have been suggested to affect the response to adjuvant chemotherapy, whereas three target genes have been associated with the response to adjuvant endocrine therapy. The latter included two of the four loci (*ASPH* and *GATA3*) associated with age-dependent survival effect in *BRCA2* carriers. Seven genes from six loci had been suggested to be involved in the regulation of immune response. However, four of these genes were also associated with regulation of mammary cell differentiation.

### Comparison of the results to survival associations from a general breast cancer population

None of the survival associations discovered in the *BRCA1/2* carriers were detected in the BCAC data of unselected and familial non-*BRCA1/2* breast cancer patients, when our results were compared to those of Escala-Garcia et al. (Supplementary Table 7)^23^.

## Discussion

We studied germline genetic variants associated with the survival of breast cancer patients carrying pathogenic variants in the high-risk *BRCA1* and *BRCA2* genes. We identified one variant, rs57025206, which was highly significantly associated with poor survival of *BRCA1* carriers with ER-negative breast cancer, with HR = 4.37, 95% CI 3.03–6.30, *P* = 3.1 × 10^−9^. Furthermore, we discovered 17 additional candidate loci, which could enhance the understanding of the biological processes modifying the survival of breast cancer patients and form a basis for future research. It was noteworthy that the single significant discovery was made in a subgroup of women with *BRCA1* pathogenic variants and ER-negative breast cancer, and this was attenuated in the wider analysis of all *BRCA1* carriers, because inclusion of the ER-positive breast cancer cases, where the association did not exist, diluted the effect in the combined analysis. Thus, the strength of the discovery analysis came from a phenotypically homogeneous group of cases with shared disease etiology, rather than a large number of study subjects.

Minor alleles of two rare variants, rs9824779 and rs56942057, are in complete linkage (*D*′ = 1.0, *R*^2^ = 0.036) with the rare, poor-survival, allele of rs57025206. These two variants affect conserved transcription factor binding sequences in enhancers targeting the *DCAF1* TSS, and the enhancer–TSS interaction is present in HMEC (Fig. 2). Based on our analysis, these variants would be the best functional candidates causing the association signal, although their frequency was too low to allow survival analysis in the CIMBA data.

*DCAF1* is an ubiquitin ligase substrate receptor, which recognizes and binds substrates for the CLR4 and HECT-type ubiquitin ligases, thus regulating the substrate half-life. Furthermore, the casein kinase-like and Lis1 homology domains of the DCAF1 protein have been shown to modulate histones by phosphorylation and deacetylation, respectively^37,38^. According to in vitro models, DCAF1 silencing reduced proliferation and colony formation of MCF7 breast cancer and DU145 prostate cancer cell lines^39,40^, whereas high DCAF1 expression blocked the expression of tumor suppressor genes via H2A phosphorylation, and induced a proliferative gene expression signature together with FOXM1 (refs ^38,40^). In mammary epithelial progenitor cells, DCAF1 is involved in the regulation of the Hippo pathway and ER-alpha, contributing to the maturation of luminal and basal lineages^41^.

The genomic locus of rs57025206, 3p21, is frequently deleted in mammary carcinomas, especially in ER-negative and high-grade tumors^42–44^. Subsequent research has not been able to name an unequivocal cancer driver gene, even though several candidates have been suggested, including *BAP1* and *MIR135A*^45,46^, which were detected as eQTL variants in our target gene analysis (Supplementary Data 2). However, the topological data suggested that these eQTLs might represent tissue-specific regulatory associations not present in HMEC, and that if the rs57025206-related survival effect is mediated via *BAP1* or *MIR135A*, it probably reflects host–tumor interactions.

To explore the molecular mechanisms possibly contributing to the survival of breast cancer patients with pathological *BRCA1* and *BRCA2* variants, we included in a target gene analysis and functional literature review 17 sub-genome-wide significant variants with reasonably low false discovery probability. Four variants came from the *BRCA1*–*BRCA2* meta-analysis, four variants had age-dependent survival association for *BRCA2* carriers, and the remaining nine variants were associated with the survival of *BRCA1* carriers (Tables 2–4). With the selected thresholds for reporting, five to six loci are expected to be false discoveries, while the rest are likely to be true survival loci. Significant between-strata heterogeneity was not detected for any tested variant.

A literature review of the plausible target genes indicated that similar biological functions underscored the genetic survival associations of *BRCA1* and *BRCA2* carrier breast cancer patients (Supplementary Table 6 and Fig. 3). The most prevalent function was the regulation of epithelial cell proliferative and migratory capacities, suggesting that germline variants modifying the liability of phenotypic changes in epithelial cells could be key determinants for the outcome of breast cancer. Another interesting observation was the accumulation of genes regulating mammary epithelial cell differentiation and stemness on the loci associated with the survival of *BRCA1* carriers with ER-negative breast cancer. In addition to *DCAF1*, described above, these candidate genes include *ZRANB1* and *CTBP2* located on 10q26.13. *ZRANB1* is a deubiquitinase targeting, e.g., EZH2, a component of the PRC2 and direct regulator of *BRCA1* activation^47,48^, whereas *CTBP2* is a transcriptional corepressor priming target gene promoters, including *BRCA1*, for PRC2-dependent silencing^49,50^.

The functional review highlighted treatment response as another important mechanism contributing to the survival of *BRCA1/2* pathogenic variant carriers. The *GATA3* locus from the analysis of *BRCA2* carriers, the *TPM2/GBA2* locus from the *BRCA1–BRCA2* meta-analysis, and five out of ten loci from the analyses of *BRCA1* carriers have previously been reported to affect the response to adjuvant chemotherapy (Supplementary Table 6 and Fig. 3). Interestingly, *KIF26B, GAS7, MIR135A*, and *CTBP2*, all potential target genes of variants associated with the survival of *BRCA1* carriers, have been suggested to modify the response to platinum-based chemotherapy^51–56^. Furthermore, *CTBP2* has been shown to affect the sensitivity to PARP inhibitors by targeting the *BRCA1* promoter for epigenetic silencing^50^. Platinum compounds and PARP inhibitors are effective agents for adjuvant therapy of *BRCA1* carriers^57,58^. Therefore, the information on genetic variation in these potential response-modifier loci could add to design of targeted therapy trials.

In the analysis of *BRCA2* carriers, we did not find any variants exceeding the discovery threshold. This may be partly explained by the smaller number of *BRCA2* carriers, and consequently events, in comparison to *BRCA1* carrier analysis. However, it was intriguing that with similar thresholds for *P*-value and false discovery probability, we discovered four variants with age-dependent survival effects. The majority of the *BRCA2*-related breast cancers are hormone receptor positive (Table 1), and rely on the endogenous supply of estrogen and possibly progesterone for proliferation^59^. The level of both of these steroid hormones decreases between the ages of 35 and 55 years^60^, consistent with the diagnosis age of the majority of the *BRCA2* carrier cancers in these data (Table 1). The target gene functional review indicated that two loci with age-dependent survival effect in *BRCA2* carriers, *GATA3* and *ASPH*, are associated with differences in response to adjuvant endocrine treatment^61,62^, further supporting the hypothesis that in hormone receptor-positive breast cancer, the age-related differences in hormone secretion and sensitivity may affect the survival of the patients.

We can draw two conclusions from our analyses, which support the hypothesis that the tumor etiology and subtype influence the genetic survival associations. First, the strongest *BRCA1* survival variants were not associated with the survival of *BRCA2* variant carriers. Second, we did not find any corroboration for our discoveries from the BCAC data (Supplementary Table 7). *BRCA1* and *BRCA2* carrier tumors have characteristic mutation profiles, accompanied by homologous recombination deficiency, which distinguishes these from unselected breast carcinomas on a molecular level^25^. Notwithstanding, the mutations in these two genes are associated with different breast cancer subtypes^26,27^. Taken together, our results encourage accuracy in definition of the outcome of interest and stringency in collecting comparable study subjects, in order to improve the future survival studies.

Like many earlier genetic survival association studies, this study had a limited discovery power, despite the fact that the analyzed high-risk variant carrier cohorts were enriched with poor-prognosis cancers, and therefore contained more events than any equally sized cohort of unselected patients would contain. With two phenotypically coherent groups of breast cancer patients, which represent the largest collections of *BRCA1* and *BRCA2* pathologic variant carriers in the world, we were able to identify a single genome-wide significant locus, but were probably underpowered to detect loci with more modest effect sizes. We estimate that better coverage of treatment and pathology would have greatly improved our analyses, especially as the functional literature review suggested that the survival differences could be mediated by differential response to adjuvant treatment. Unfortunately, we did not have enough well-documented treatment data available to test the hypothesis. The retrospective nature of our data brought its own limitations. The data included a notable proportion of prevalent cases, enrolled into the participating studies after a prolonged time period after the initial breast cancer diagnosis. The choice of the overall survival as the basis of the analyses impairs the clinical interpretation of the results to some degree. However, most of the follow-up information came from registries, and therefore the all-cause death was the best available indicator for poor prognosis. We tried to overcome these shortcomings with appropriate analysis methods for the prevalent data, with posterior likelihood estimation and sensitivity analyses with breast cancer-specific death as an end point, as well as by testing for internal consistency.

In conclusion, we report a survival locus for *BRCA1* carriers with ER-negative breast cancer. Furthermore, the results from the exploratory analyses suggest that the survival in women with breast cancer is influenced by a complex action between clinical characteristics, tumor biology, and the germline genetic landscape.

## Methods

### Study subjects

The study subjects included women of European ancestry diagnosed with invasive breast cancer before the age of 70 years, enrolled in studies participating in the CIMBA (Supplementary Table 1). A CIMBA study was included in the analyses if it provided sufficient amount of follow-up data, defined as at least 15 study subjects at risk during the time when five events occurred. The study-wise inclusion criterion was applied separately for the main analyses and the ER-specific subgroup analyses. This selection yielded survival data on 3008 women carrying pathogenic germline variants in *BRCA1* from 21 studies and 2009 women carrying variants in *BRCA2* from 15 studies. Tumor characteristics of the study subjects are provided in Table 1. The *BRCA1* carriers were collected from 2664 families: 2391 families with one study subject, 220 families with two study subjects, and 53 families with more than two study subjects. The *BRCA2* carriers were collected from 1713 families: 1486 families with one study subject, 182 families with two study subjects, and 49 families with more than two study subjects. All participating studies were approved by their appropriate ethics review boards, and all subjects provided informed consent.

### Genotype data

The study subjects were genotyped with a custom-made array as a part of the OncoArray project^31^. Details on the variant selection and data quality control have been published elsewhere. In short, the genotyping array included a GWAS backbone (Illumina HumanCore) and potentially cancer-associated variants nominated by the six participating consortia. Genotyping of the CIMBA samples was conducted in six independent laboratories, which used the same HapMap reference samples and a common genotype-clustering file to ensure interlaboratory comparability^31^. The data were imputed using the 1000 genomes as a reference panel, as described previously^32^. In the analyses, we included variants with at least 60 carriers, corresponding to MAFs 0.01 and 0.015 for *BRCA1* and *BRCA2* carriers, respectively. This yielded data on ~9.7 million SNPs for *BRCA1* carriers and 9.1 million SNPs for *BRCA2* carriers.

### Survival analysis

The patients were followed from the diagnosis of the first primary breast cancer until death of any cause and censored after 15 years or when lost from follow-up. Left truncation was applied to account for delayed study entry. In the *BRCA1* carrier analysis, the number of person years was 16,056, the number of deaths 461, and the 15-year survival rate 0.66. The maximum number of study subjects at risk was 1336 and this was reached 2.8 years after the baseline. The *BRCA2* carrier dataset covered 10,712 person years with 311 deaths leading to a 15-year survival rate of 0.65. Here, the highest number of study subjects at risk, 930, was reached 3.9 years after the baseline.

The genome-wide analysis of association between genetic variants and all-cause mortality was performed with Cox regression as implemented in the *survival* library of the R environment for statistical computing version 3.5.1 and 3.6.0 (refs ^63–66^). Linear per-allele risk model and dominant inheritance model were estimated in parallel. Analyses were adjusted for diagnosis age, allowing for variant–age interaction, and stratified by country group to account for population-specific genetic and clinical features (Supplementary Table 1). Likelihood-ratio test was used as a measure of significant association with alpha risk *P* < 5 × 10^−7^ (two-sided). The interaction model, i.e., Cox regression model including an interaction term, coded as a product of the number of the effect alleles (0, 1, 2) and diagnosis age (continuous), was tested against a nested model containing the variant and age without interaction. Robust variance estimation was used to account for relatedness of study subjects from the same families. Parallel genome-wide analyses were performed also within the biologically homogeneous patient groups: *BRCA1* carriers with ER-negative tumors (1385 patients from 17 studies, see above the study-wise inclusion criterion for study-stratified analysis) and *BRCA2* carriers with ER-positive tumors (1050 patients from 14 studies). The results from analysis of all *BRCA1* were compared to results from analysis of all *BRCA2* carriers with Pearson correlation, and combined using a fixed-effects meta-analysis as implemented in R library *metafor*^67^. For the meta-analysis, the standard errors were recalculated using the likelihood-ratio test statistic to avoid inflation caused by rare variants, as suggested previously^23^.

Using R-library *powerSurvEpi*^68^, we estimated that we had sufficient statistical power, with the selected alpha-risk (5 × 10^−7^) and beta-risk 0.2, to detect significant risk associated with common variants (MAF > 0.10), if the HR was >1.8, whereas for rare variants (MAF 0.03–0.10), the HR should be >2.5 for a discovery. Since we selected an alpha-level lower than the commonly accepted genome-wide significance threshold 5 × 10^−8^, we calculated a BFDP with R library *gap* for nominal variant effects and FDR for the interaction models to estimate the validity of our findings^69–72^. In the BFDP analysis, the prior discovery probability was set to 0.0001 and HR alternatively to 1.3, 1.8, or 2.5, as in Escala-Garcia et al.^23^, and according to the estimated thresholds for discovery from the power analyses. SNPs with BFDP or FDR less than one-in-three were considered as interesting discoveries.

We performed additional survival analyses for the newly discovered SNPs only. These included multivariable survival model adjusted for tumor pathologic characteristics and analysis of breast cancer-specific death. Complete pathologic data were available for 1104 (36.7%) *BRCA1* and 743 (37.0%) *BRCA2* carriers, and the data on breast cancer-associated death for 2066 (68.7%) *BRCA1* and 1591 (79.2%) *BRCA2* carriers. However, data on both pathology and cause of death were available only for 683 (22.7%) *BRCA1* and 544 (27.1%) *BRCA2* carriers. The pathologic covariates in the multivariable model were coded as follows: tumor ER expression: categorical—no expression/positive expression (not included in the analyses of ER-stratified patient groups); tumor PgR expression: categorical—no expression/positive expression; tumor grade: linear—1, 2, 3; tumor size: linear—1 = less than 2 cm in diameter, 2 = diameter between 2 cm and 5 cm, 3 = larger than 5 cm in diameter; and lymph node status: categorical—affected/not affected. The validity of the proportional hazards assumption was evaluated for all discovered variants.

### Candidate gene identification

The newly discovered survival SNPs were characterized in silico utilizing data from the 1000 genomes^35^ and Encode^36^ projects as integrated in databases LDlink^73,74^, RegulomeDB^75^, and GeneCards^76,77^. We retrieved all short genomic variants linked with the discovered survival variants in the 1000 genomes European data with *D*′ > 0.8. The proxy variants were subjected to RegulomeDB analysis and all variants with scores 1a–2f were considered as regulatory variants potentially contributing to the survival signal (Supplementary Data 1). The variant positions were matched to locations of protein-coding genes and validated enhancer regions to identify the plausibly target genes. Furthermore, we retrieved eQTL genes from GTEx^78,79^ releases V6, V6p, V7, and Westra et al. blood eQTL dataset^80^ for all functional proxy SNPs (*D*′ > 0.8) and for all strongly linked proxy SNPs (*R*^2^ > 0.8) irrespective of their functional annotation (Supplementary Data 2). The topological chromatin status at the rs57025206 locus was investigated in Rao et al.^81^ data from HMEC using the 3D genome browser^82^.

For a functional summary, we performed a systematic literature search using the gene symbol and any of the keywords “breast cancer”, “mammary”, “estrogen”, “immune”, “leukocyte”, and “lymphocyte”. If none of these queries returned relevant publications, the search was continued with gene symbol and “cancer” or with gene symbol alone. This was the first stage of literature search. In the second stage, the queries consisted of the gene symbol and any of the recurrent functional terms or interacting proteins detected in the first stage (Fig. 3).

The survival associations of the candidate genes’ mRNA expression were tested in the KM plotter database for breast cancer (http://kmplot.com/analysis/)^83^, restricting the analysis in the relevant group of mammary tumors: ER-negative tumors for target genes from the analyses of *BRCA1* carriers, ER-positive tumors for genes from *BRCA2* analysis, and all tumors for genes from the meta-analysis. The best cutoff for the categorical survival analysis was selected automatically and results with FDR ≤ 5% were reported. Furthermore, the linear association between candidate genes’ mRNA expression and patient survival was tested in the METABRIC data (EGAD00010000434, 1302 breast cancer patients) using Cox regression. Expression data were available for 29 of the 39 candidate genes. For each of the candidate genes, the samples were split into three categories based on 33 and 67% percentiles of the expression values, and the categories analyzed for 10-year overall survival. The results are included in the functional summary in Supplementary Table 6.

### Reporting summary

Further information on research design is available in the Nature Research Reporting Summary linked to this article.

## Supplementary information

Supplementary Information

Data Set 1

Data Set 2

Reporting summary checklist

## Data Availability

All summary results will be made available on the CIMBA website upon publication (http://cimba.ccge.medschl.cam.ac.uk/). A subset of the genotype data that support the findings of this study is publically available via dbGaP: https://identifiers.org/dbgap:phs001321.v1.p1. Requests for data can be made to the CIMBA Data Access Coordination Committee. DACC approval is required to access data from the BCFR-ON, EMBRACE, GC-HBOC, HEBCS, HEBON, IHCC, IPOBCS, MCGILL, and OUH studies (Supplementary Table 1). The contact for data access requests is Lesley McGuffog (ljm26@medschl.cam.ac.uk), Data Manager, Department of Public Health and Primary Care, University of Cambridge. A full description of data supporting the findings of this study is available in figshare: 10.6084/m9.figshare.12613043 (ref. ^84^).

## Code availability

All statistical analyses were performed within the R environment for statistical computing version 3.5.1 and 3.6.0, using libraries *survival*, *gap*, *metafor*, and *powerSurvEpi*, for Cox regression, BFDP, meta-analysis, and power estimation, respectively.
