# Supplementary material for: Association of germline variation with the survival of women with BRCA1/2 pathogenic variants and breast cancer
Source: NPJ Breast Cancer. 2020 Sep 10;6:44. doi: 10.1038/s41523-020-00185-6 (PMC7483417; doi:10.1038/s41523-020-00185-6)
Supplement: Supplementary file 1 — Supplementary Information [file 41523_2020_185_MOESM1_ESM.pdf]

Association of germline variation with the survival of women with *BRCA1/2* pathogenic variants and breast cancer

Taru A. Muranen, Sofia Khan, Rainer Fagerholm, Kristiina Aittomäki, Julie M. Cunningham, Joe Dennis, Goska Leslie, Lesley McGuffog, Michael T. Parsons, Jacques Simard, Susan Slager, Penny Soucy, Douglas F. Easton, Marc Tischkowitz, Amanda B. Spurdle, kConFab Investigators, Rita K. Schmutzler, Barbara Wappenschmidt, Eric Hahnen, Maartje J. Hooning, HEBON Investigators, Christian F. Singer, Gabriel Wagner, Mads Thomassen, Inge Sokilde Pedersen, Susan M. Domchek, Katherine L. Nathanson, Conxi Lazaro, Maria Rossing, Irene L. Andrulis, Manuel R. Teixeira, Paul James, Judy Garber, Jeffrey N. Weitzel, SWE-BRCA Investigators, Anna Jakubowska, Drakoulis Yannoukakos, Esther M. John, Melissa C. Southey, Marjanka K. Schmidt, Antonis C. Antoniou, Georgia Chenevix-Trench, Carl Blomqvist, Heli Nevanlinna

Supplementary Information

Supplementary Figure 1. The survival effect associated with rs57025206 in all *BRCA1* carriers………… 2

Supplementary Figure 2. Manhattan plots………… 3

Supplementary Figure 3. Plots for survival variants discovered in *BRCA1* carriers………… 4

Supplementary Figure 4. Plots for survival variants discovered in the meta-analysis………… 5

Supplementary Figure 5. Plots for survival variants discovered in *BRCA2* carriers………… 6

Supplementary Table 1. Description of CIMBA studies………… 7

Supplementary Table 2. Additional models for *BRCA1* carriers………… 8

Supplementary Table 3. Additional meta-analysis models………… 8

Supplementary Table 4. Additional models for *BRCA2* carriers………… 9

Supplementary Table 5. Survival associations of variants with age-dependent survival effect in *BRCA2* carriers, stratified by the tumor ER-status………… 9

Supplementary Table 6. Literature-based functional annotation of the target genes………… 10

Supplementary Table 7. Survival associations in the Breast Cancer Association Consortium (BCAC) data………… 14

Supplementary References………… 15

Supplementary note………… 19

Supplementary Data 1. Identification of potential target genes based on variant position and functional annotations………… 21

Supplementary Data 2. eQTL-analysis in GTEx and Westra et al. data………… 21

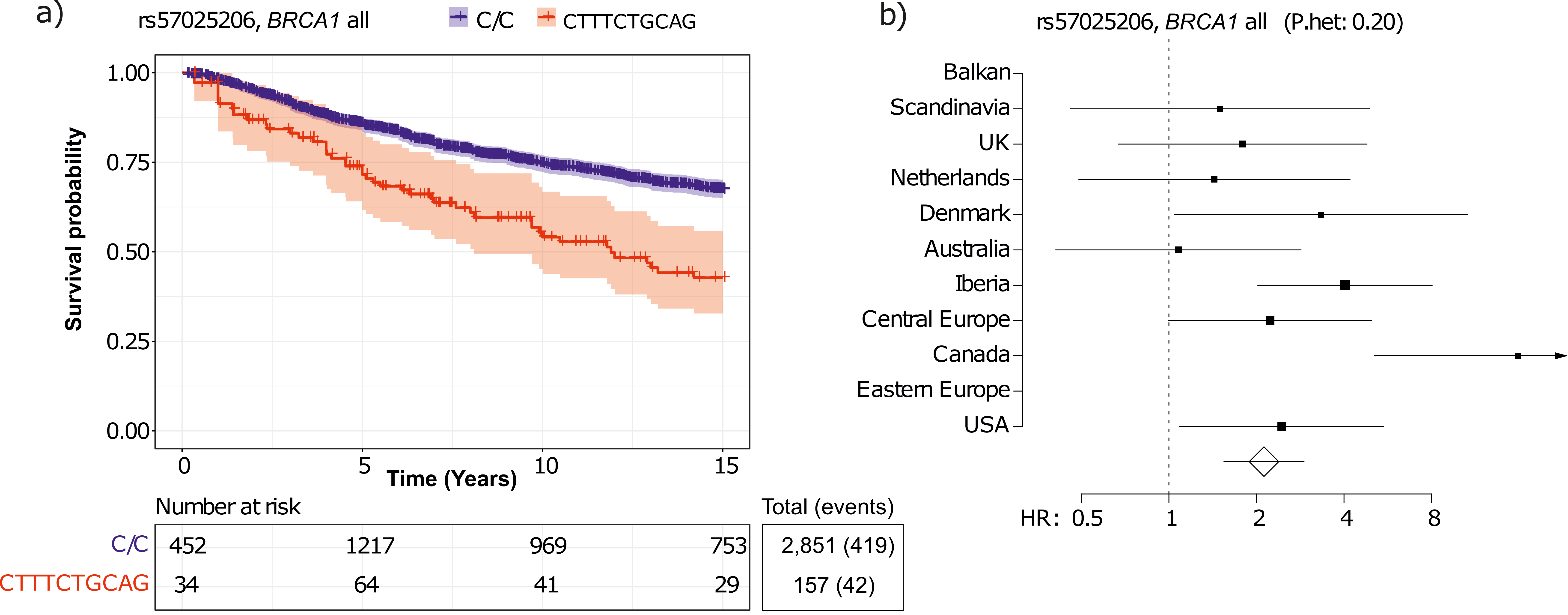

Supplementary Figure 1. The survival effect associated with rs57025206 in all *BRCA1* carriers.
a) Kaplan-Meier plot stratified by rs57025206. b) Forest plot of hazard ratios (HR) accross country groups.
P.het: P-value against between-study heterogeneity.

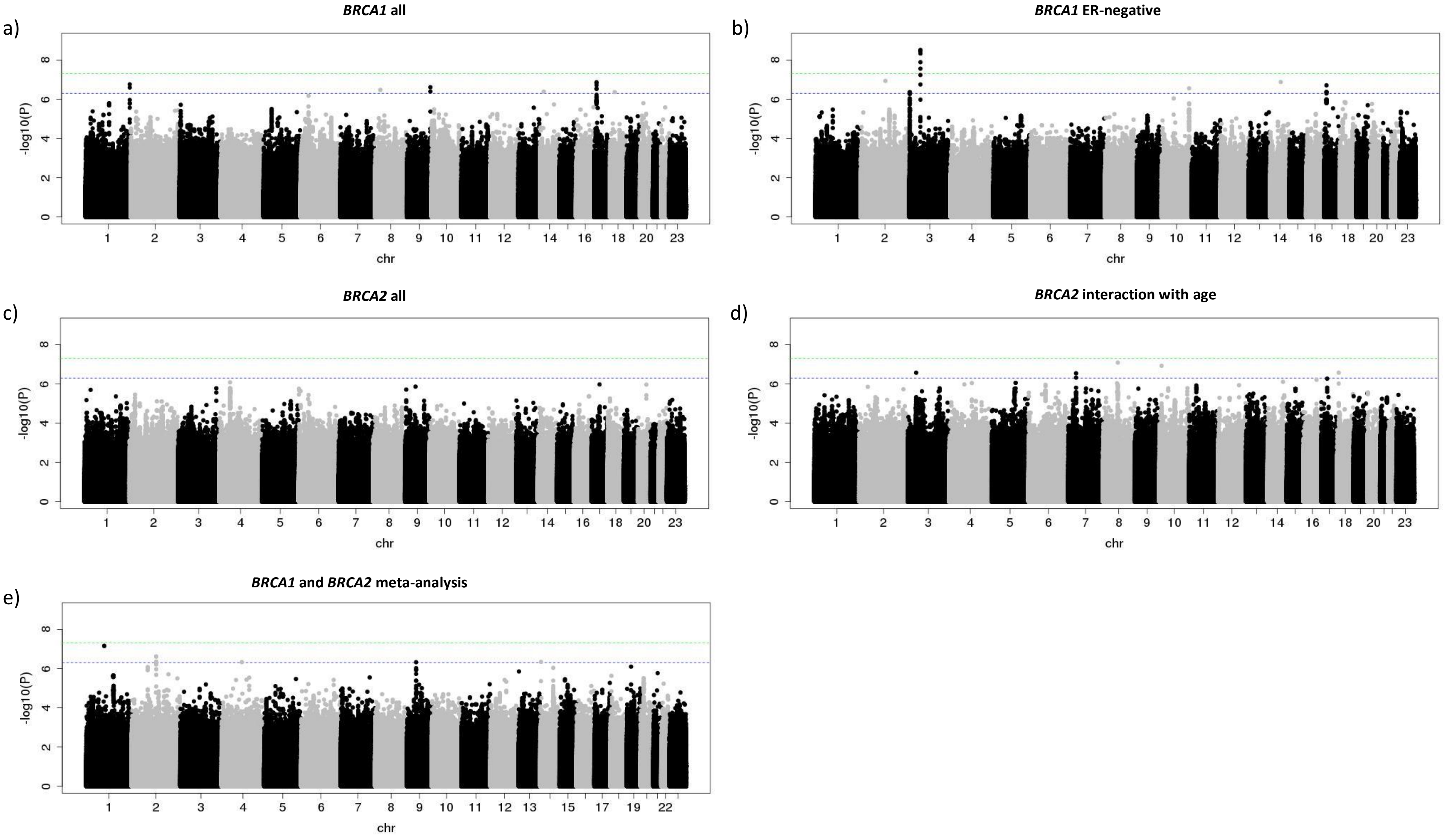

Supplementary Figure 2. Manhattan plots.

Manhattan plots from survival-association analyses of a) *BRCA1* carriers, b) *BRCA1* carriers with ER-negative breast cancer, c) *BRCA2* carriers, d) *BRCA2* carriers from variant-diagnosis age interaction analysis, and e) a meta-analysis of nominal survival associations in *BRCA1* and *BRCA2* carriers. P-values from likelihood ratio test plotted against genomic position. (Green dashed line: $5 \cdot 10^{-8}$; blue dashed line: $5 \cdot 10^{-7}$)

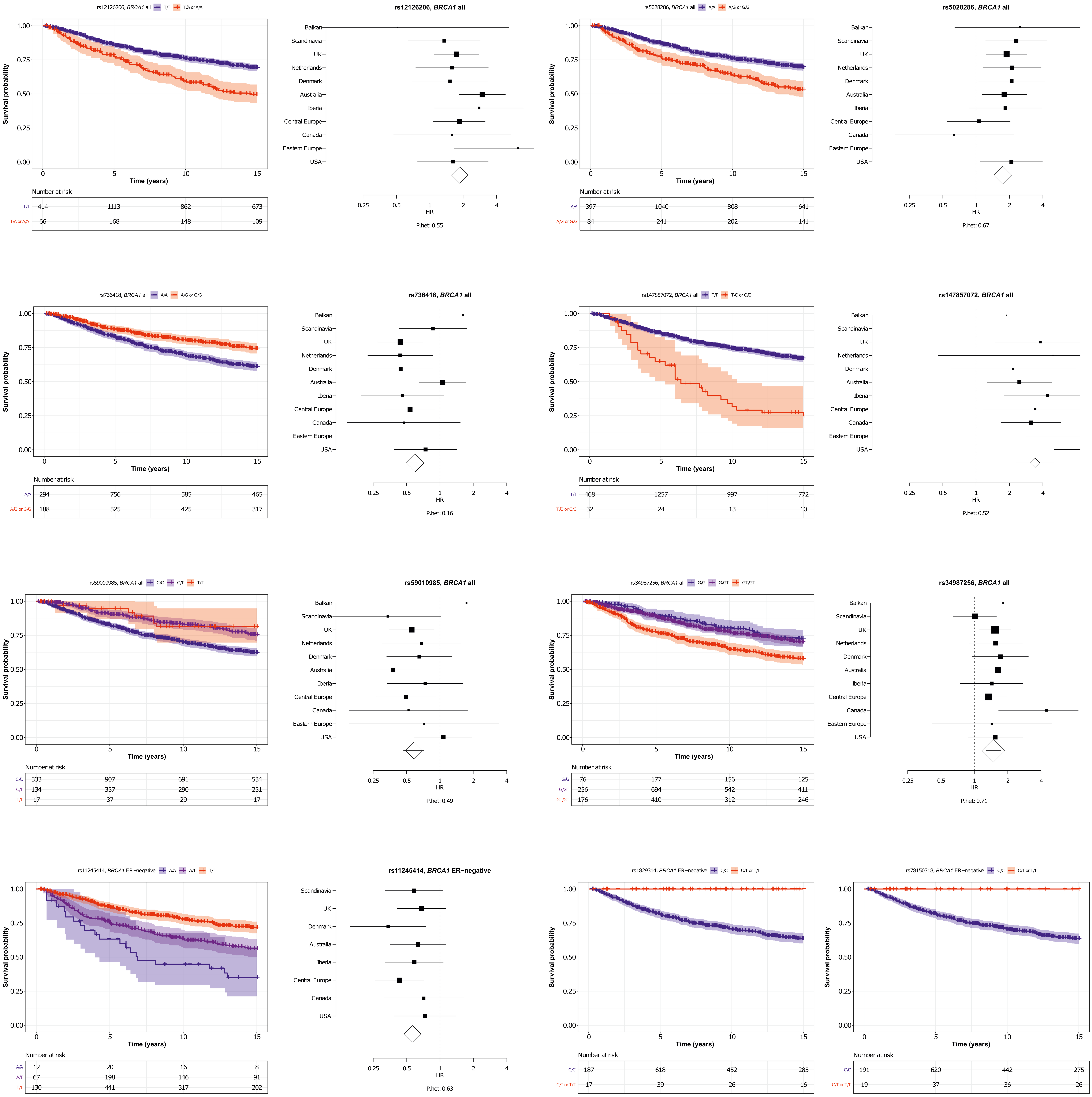

Supplementary Figure 3. Plots for survival variants discovered in *BRCA1* carriers
Kaplan-Meier curves graphically presenting the proportion of surviving patients during the 15 years after primary breast cancer diagnosis in the discovery data (see Table 2), stratified by the 10 *BRCA1*-associated survival variants (1st and 3rd panel from left). Forest plots presenting the effect sizes in different country groups (2nd and 4th panel). Because the carriers of effect alleles of rs1829314 and rs78150318 had no events, the forest plots could not be drawn.

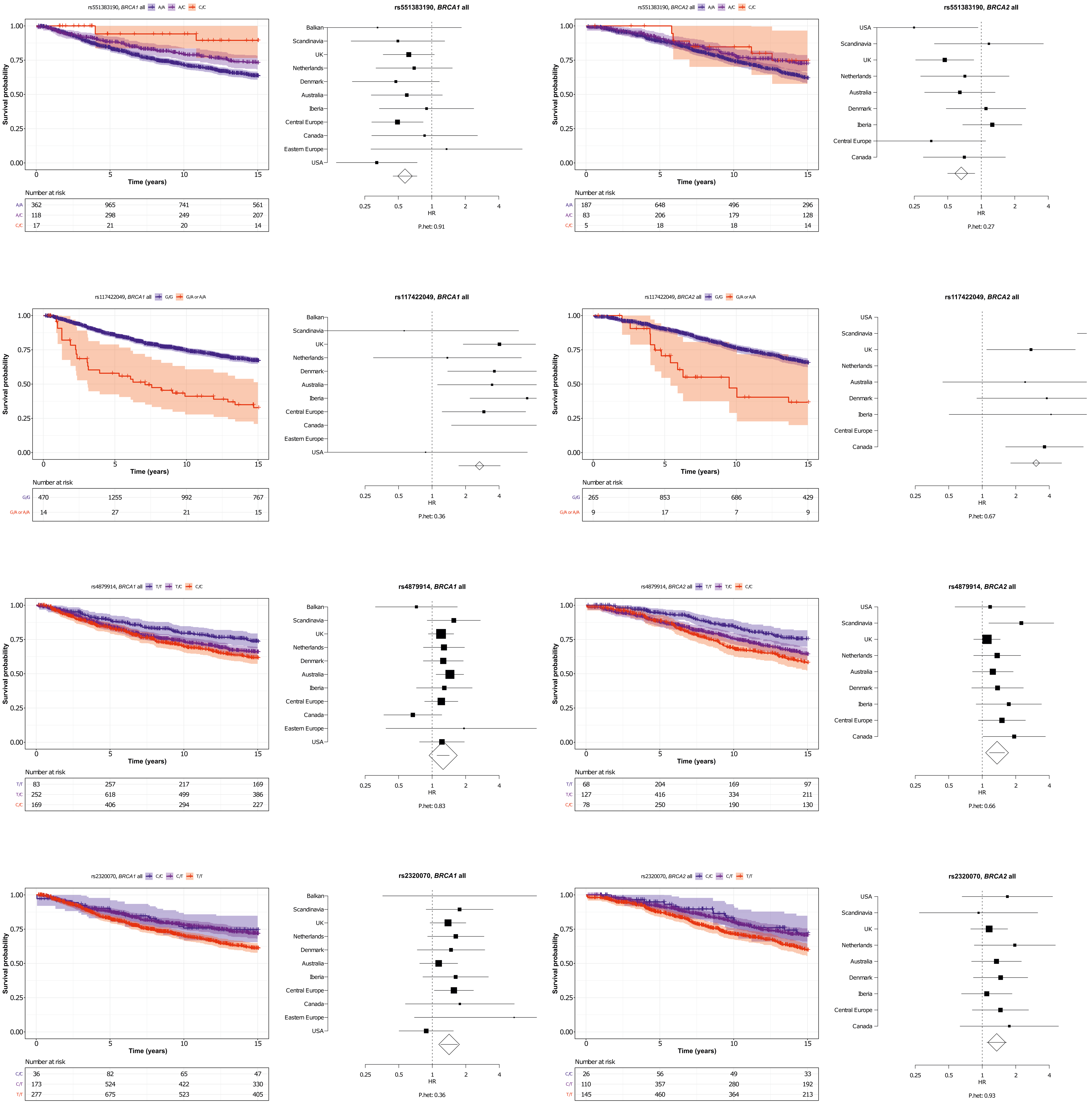

Supplementary Figure 4. Plots for survival variants discovered in the meta-analysis
Kaplan-Meier curves stratified by the four variants with consistent survival effects for *BRCA1* and *BRCA2* carriers (1st and 3rd panel from the left, respectively). Forest plots presenting the effect sizes in different country groups (2nd and 4th panel).

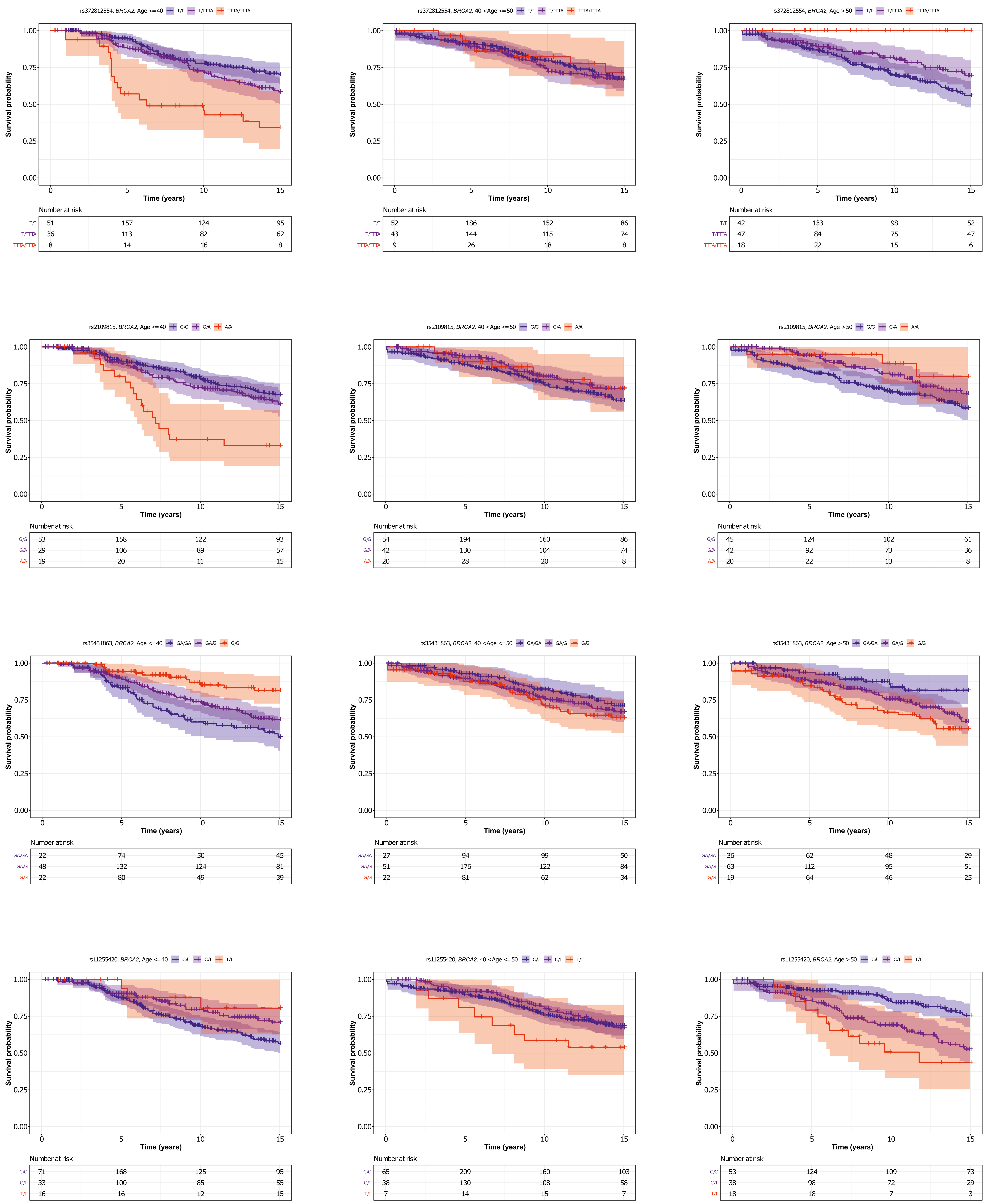

Supplementary Figure 5. Plots for survival variants discovered in *BRCA2* carriers
Kaplan-Meier curves stratified by the four variants with age-dependent survival effect for *BRCA2* carriers in three age-groups: patients diagnosed before the age of 40 years (left), between the age of 40 and age of 50 years (middle), and after the age of 50 years (right).

Supplementary Table 1. Description of CIMBA studies.

Study acronym	Study name	Country	Country group	Study subject ascertainment	Number of study subjects		Number of events	
					BRCA1	*BRCA2*	*BRCA1*	*BRCA2*
BCFR-AU	Australian site of the Breast Cancer Family Registry	Australia	Australia	Population based	25		8	
BCFR-NC	Northern California site of the Breast Cancer Family Registry	USA	USA	Population based	31		6	
BCFR-ON/ OCGN	Ontario site of the Breast Cancer Family Registry/ Ontario Cancer Genetics Network	Canada	Canada	Clinic and population based	73	53	15	17
CBCS	Copenhagen Breast Cancer Study	Denmark	Denmark	Clinic based	73	63	14	9
COH	City of Hope Cancer Center	USA	USA	Clinic based	101		15	
DEMOKRITOS	National Centre for Scientific Research Demokritos	Greece	Balkan	Clinic based	57		9	
DFCI	Dana Farber Cancer Institute	USA	USA	Clinic based	64	46	8	8
EMBRACE	Epidemiological Study of Familial Breast Cancer	UK	UK	Clinic based	694	678	95	89
GC-HBOC	German Familial Breast Group	Germany	Central-Europe	Clinic based	368	207	33	17
HEBCS	Helsinki Breast Cancer Study	Finland	Scandinavia	Clinic based	62	61	18	16
HEBON	Hereditary Breast and Ovarian cancer study the Netherlands	Netherlands	Netherlands	Clinic based	234	93	41	28
ICO	Institut Català d'Oncologia	Spain	Iberia	Clinic based	87	100	14	16
IHCC	International Hereditary Cancer Centre	Poland	East-Europe	Clinic based	94		7	
IPOBCS	Portuguese Oncology Institute-Porto Breast Cancer Study	Portugal	Iberia	Clinic based	39	82	12	11
KCONFAB	Kathleen Cuningham Consortium for Research into Familial Breast Cancer	Australia	Australia	Clinic based	331	245	53	37
MUV	General Hospital Vienna	Austria	Central-Europe	Clinic based	218	108	38	17
OUH	Odense University Hospital	Denmark	Denmark	Clinic based	172	143	30	27
SWE-BRCA	Swedish Breast Cancer Study	Sweden	Scandinavia	Clinic based	97		19	
UPENN	University of Pennsylvania	USA	USA	Clinic based	118	82	13	11
VFCTG	Victorian Familial Cancer Trials Group	Australia	Australia	Clinic based	70	48	13	8

Supplementary Table 2. Additional models for BRCA1 carriers.

Variant effects in multivariate and in breast cancer-specific survival analyses for the SNPs, which were considered novel discoveries in the analysis of *BRCA1* carriers and *BRCA1* carriers with ER-negative breast cancer.

SNP	Analysis subgroup	Genetic model	HR for all-cause mortality in a model adjusted for tumor characteristics		Nominal HR for breast cancer-specific death		HR for breast cancer-specific death in a model adjusted for tumor characteristics	
			HR	[95% CI]	HR	[95% CI]	HR	[95% CI]
rs12126206	BRCA1 all BC	dominant	1.96	[1.37-2.81]	1.53	[1.08-2.17]	1.91	[1.07-3.39]
rs5028286	BRCA1 all BC	dominant	1.55	[1.11-2.17]	1.44	[1.04-1.99]	1.21	[0.69-2.10]
rs736418	BRCA1 all BC	dominant	0.60	[0.43-0.83]	0.72	[0.53-0.96]	0.41	[0.24-0.70]
rs147857072	BRCA1 all BC	dominant	4.95	[2.67-9.18]	2.55	[1.41-4.61]	3.32	[1.28-8.58]
rs59010985	BRCA1 all BC	per-allele linear	0.49	[0.34-0.70]	0.61	[0.44-0.83]	0.66	[0.40-1.09]
rs34987256†	BRCA1 all BC	per-allele linear	1.44	[1.09-1.89]	1.68	[1.31-2.16]	1.61	[1.04-2.48]
rs1829314	BRCA1 ER-negative	dominant	0.00	[0.00-0.00]	0.00	[0.00-0.00]	0.00	[0.00-0.00]
rs57025206	BRCA1 ER-negative	per-allele linear	6.19	[3.73-10.3]	4.68	[2.81-7.80]	4.98	[1.91-13.0]
rs11245414	BRCA1 ER-negative	per-allele linear	0.68	[0.51-0.91]	0.54	[0.40-0.72]	0.63	[0.41-0.97]
rs78150318	BRCA1 ER-negative	dominant	0.00	[0.00-0.00]	0.00	[0.00-0.00]	0.00	[0.00-0.00]

†The hazard associated with the SNP did not significantly violate of the proportional hazards assumption in the multivariate models or in the analyses of breast cancer-associated death.

Supplementary Table 3. Additional meta-analysis models.

Variant effects in multivariate and in breast cancer-specific survival analyses for the SNPs, which were considered novel discoveries in the *BRCA1-BRCA2*-carrier meta-analysis.

SNP	Analysis subgroup	Genetic model	HR for all-cause mortality in a model adjusted for tumor characteristics		Nominal HR for breast cancer-specific death		HR for breast cancer-specific death in a model adjusted for tumor characteristics	
			HR	[95% CI]	HR	[95% CI]	HR	[95% CI]
rs551383190	BRCA1/BRCA2	per-allele linear	0.61	[0.46-0.82]	0.63	[0.49-0.81]	0.68	[0.50-0.94]
rs117422049	BRCA1/BRCA2	per-allele linear	3.43	[0.83-14.3]	3.24	[1.97-5.32]	3.60	[2.00-6.47]
rs4879914	BRCA1/BRCA2	per-allele linear	1.40	[1.18-1.67]	1.39	[1.20-1.60]	1.49	[1.24-1.80]
rs2320070	BRCA1/BRCA2	per-allele linear	1.31	[1.06-1.63]	1.40	[1.17-1.68]	1.46	[1.02-2.09]

Supplementary Table 4. Additional models for BRCA2 carriers.

Variant effects in multivariate and in breast cancer-specific survival analyses for the SNPs, which had age-dependent survival effect for *BRCA2* carriers.

SNP	Analysis subgroup	Genetic model	HR for all-cause mortality in a model adjusted for tumor characteristics				Nominal HR for breast cancer-specific death				HR for breast cancer-specific death in a model adjusted for tumor characteristics			
			dgAge < 40 years		dgAge ≥ 40		dgAge < 40 years		dgAge ≥ 40		dgAge < 40 years		dgAge ≥ 40	
			HR	[95% CI]	HR	[95% CI]	HR	[95% CI]	HR	[95% CI]	HR	[95% CI]	HR	[95% CI]
rs372812554	BRCA2 all BC	per-allele interaction with age	2.11	[1.33-3.34]	0.84	[0.57-1.23]	1.91	[1.32-2.76]	0.74	[0.53-1.04]	2.04	[1.09-3.85]	0.66	[0.39-1.12]
rs2109815	BRCA2 all BC	per-allele interaction with age	1.70	[1.09-2.67]	0.92	[0.64-1.32]	1.78	[1.24-2.56]	0.87	[0.64-1.18]	2.01	[1.17-3.46]	1.12	[0.66-1.91]
rs35431863	BRCA2 all BC	per-allele interaction with age	0.58	[0.29-1.14]	1.29	[0.94-1.78]	0.55	[0.38-0.78]	1.27	[0.98-1.65]	0.62	[0.27-1.44]	1.40	[0.94-2.09]
rs11255420	BRCA2 all BC	per-allele interaction with age	0.62	[0.32-1.18]	1.28	[0.87-1.90]	0.67	[0.44-1.01]	1.73	[1.28-2.33]	0.44	[0.19-1.05]	1.29	[0.74-2.24]

Supplementary Table 5. Survival associations of variants with age-dependent survival effect in BRCA2 carriers, stratified by the tumor ER-status.

SNP	ER-positive BRCA2 carriers (n:1067)		ER-negative BRCA2 carriers (n: 302)	
	under 40 years HR [95% CI]	over 40 years HR [95% CI]	under 45 years HR [95% CI]	over 45 years HR [95% CI]
rs372812554	1.67 [1.19 - 2.36]	0.69 [0.49 - 0.98]	1.43 [0.50 - 4.05]	1.40 [0.77 - 2.54]
rs2109815	1.86 [1.24 - 2.79]	0.75 [0.56 - 1.01]	1.57 [0.84 - 2.93]	0.46 [0.20 - 1.05]
rs35431863	0.54 [0.37 - 0.80]	1.48 [1.15 - 1.90]	0.97 [0.47 - 2.02]	1.31 [0.71 - 2.41]
rs11255420	0.59 [0.41 - 0.83]	1.55 [1.24 - 1.93]	0.47 [0.15 - 1.46]	0.86 [0.43 - 1.69]

Supplementary Table 6. Literature-based functional annotation of the target genes.

Gene	Function		Ref.
KIF26B	silencing in breast cancer cell lines	reduces colony-formation	1, 2
		reduces migration and invasion	
		leads to increased E-cadherin expression and reduced N-cadherin expression	
		induces apoptosis	
		aggregates cells in G0/G1 phase	
	overexpression in breast cancer cell lines	increases proliferation (increased cell count)	2
		promotes migration and invasion	
		positively affects FGF2 expression and secretion	
		leads to MAPK/ERK pathway activation	
	knock-down in a xenograft model	reduces tumor formation	
		reduces the frequency of lung metastases	
	mRNA expression in mammary tumors	is elevated in comparison to adjacent normal	1, 2
	protein expression in mammary tumors	is elevated in comparison to adjacent normal	2
	mRNA expression in breast cancer cell lines	is elevated in comparison to MCF10A non-malignant mammary cells	1
	high expression in mammary tumors	is associated with larger, ER-positive, higher-grade, node-positive tumors, and poor survival	3
	enhances microtubule stabilization	required for asymmetrical cell structure organization and directional migration	4
	mediates multi-drug resistance	in osteosarcoma cells	5
SGCZ	loss in mammary tumors	is more frequent in early-onset tumors (METABRIC)	6, 7
		is associated with poor prognosis	
		does not affect SGCZ mRNA expression in mammary tumors, which is generally very low	
MIR383	is located in intron 1-2 of SGCZ	and MIR383 expression is coupled with SGCZ expression	8
	overexpression in mouse ovarian cells	enhances estradiol release from ovaries via repression of RMBS1, and consecutively also MYC	
	overexpression in ovarian cancer cell lines	sensitizes the cells to paclitaxel	9
RALGDS	is recruited to endosomal compartments	by RILP	10
	mediates the RILP-dependent	inhibition of cell proliferation, migration and invasion	
	serves	as a guanine nucleotide exchange factor for Ral	
	interaction with RILP	affects the MAPK/ERK pathway	
	does not directly stimulate	MAPK/ERK	11
	induces cytoskeletal reorganization	in response to FPR1 stimulation	12
MBIP	was the top-ranking breast cancer risk gene	in a genome-wide pathway analysis	13
GAS7	is hypomethylated (activated)	in ER-negative breast cancer in comparison to ER-positive breast cancer	14, 15
	transcript variant B mRNA expression	is low in mammary tumors in comparison to adjacent normal	16
		is low in early-onset tumors in comparison to late-onset tumors	
		is low in many breast cancer cell lines	
	overexpression in several breast cancer cell lines	reduces proliferation, migration and invasion	
	silencing in MCF-7 breast cancer cell line	increases proliferation, migration and invasion	
	high expression	inhibits actin polymerization via GAS7 – CYFIP1 – Rac1 – WAVE2 complex	
	is regulated by TP53		
	transcript variant B regulatory region	co-localizes with the survival variants (Supplementary Table 5)	
	rs59010985 allele T	is associated with high GAS7 expression (Table 5, Supplementary Table 6)	
	high mRNA expression is associated with	good prognosis of ER-negative breast cancer patients HR = 0.46 [0.37 - 0.58], P = 2.5E-12 (FDR 1%)	17
		good prognosis of breast cancer patients irrespective of ER-status	7
		HR per unit fold change in all 0.81 [0.72 – 0.90], P = 1.4E-4, in METABRIC data	
	mediates the apoptotic effects of	platinum compounds in hepatocellular carcinoma and neuroblastoma cells	18, 19
		gefitinib in non-small cell lung carcinoma	20

Gene	Function		Ref.
CHST9	variant rs1436904	is significantly associated with breast cancer risk in an international consortium study	21
		is associated with survival of triple-negative breast cancer patients in Chinese population	22
		is linked with the survival variant rs537497819 with D'=0.1939, R2=0.0188	
DCAF1	is an E3 ubiquitin ligase substrate receptor	which brings together the substrate and E2 ubiquitin conjugating ligase	23
	recognizes the substrate for ubiquitination	leading to proteasomal degradation of the substrate	
	substrates include	TP53 tumor suppressor, apoptosis regulator	
		ER-alpha via LATS1 interaction	24
		non-histone proteins monomethylated by EZH2 (component of the polycomb repressor complex)	25
	silencing in MCF-7 breast cancer cell line	reduces colony formation	
	regulates	DNA replication	23
		cell cycle progression	
		entry to mitosis	
		cell division	
		ER-alpha level	24
		T-cell activation induced proliferation	26
		T-cell receptor gene reorganization	
		centrosome organization by ubiquitylation as a part of the EDVP complex	27
MIR135A1	deletion in mammary tumors is associated with	higher pathologic stage	28
		younger age of onset	
		ductal histologic type	
		worse survival outcome	
		lower expression of pri-miR-135-a-1, primary precursor of mir-135A1	
	expression in mammary tumors	is higher in ER+ than ER- cancers	
	depletion in breast cancer cell lines	increases cell viability, colony formation, migration, and invasion	
		decreases sensitivity to tamoxifen treatment and contributes to acquisition of resistance	
		enhances the activity of MAPK/ERK and PI3K/AKT pathways	
	forced expression in breast cancer cell lines	decreases cell viability, colony formation, migration, and invasion	
		enhances epithelial cellular phenotype	
		suppresses the activity of MAPK/ERK and PI3K/AKT pathways	
	level is correlated	with epithelial markers	
		inversely with mesenchymal markers	
	forced expression in a mouse model	inhibits tumor formation and lung metastasis	
	promoter is bound by ER-alpha		
	directly binds and regulates ER-alpha	creating a negative feedback loop	
	expression promotes resistance to	oxaliplatin in gastric cancer	29
		gefitinib in non-small cell lung cancer	30
3p21.2 (DCAF1 and MIR135A1)	loss in mammary tumors	is more frequent than loss of any other region in 3p	31, 32
		is associated with high grade and ER-, PR- tumors	
ZRANB1	regulates	EZH2 (component of the polycomb repressor complex) by deubiquitination	33
	silencing in triple-negative breast cancer cell lines	reduces proliferation and migration	
		which can be rescued by overexpressing EZH2	
	silencing in a mouse model	suppresses tumor formation and lung metastases	
	silencing in cell lines	increases stress fibers and inhibits migration	34
	expression in normal mammary tissue	is very low	33
	high expression in mammary tumors	is associated with poor survival	
	is required for	inflammatory T-cell response	35

Gene	Function		Ref.
CTBP2	is a transcriptional co-repressor	and regulator of Golgi apparatus fission	36
	primes target genes	for repression by PRC2	37
	regulates	Aurora B kinase in early mitosis	36, 38, 39
		p16, CDH1, PTEN	
		stem-cell like characteristics of ovarian cancer cells	
	silencing in breast cancer cell lines	leads to TP53-dependent cell cycle arrest and apoptosis	36, 38, 40
		causes cell cycle stasis in aberrant mitosis	
		increases the number of binucleate cells and lagging chromosomes	
		suppresses cell migration	
	overexpression in breast cancer cell lines	increases cell proliferation and cell accumulation in S-phase	38
		decreases E-cadherin expression	
		increases vimentin and MMP2 expression	
	overexpression in a mouse xenograft model	accelerates tumor growth	
	is expressed ubiquitously in	breast cancer cell lines and patient samples	40
	high expression in mammary tumors	is associated with poor survival (ER-negative tumors: HR = 1.72 [1.39 - 2.14], P = 4.5E-7 (FDR 1%))	17, 38
	low expression in ovarian tumors	is correlated with high infiltration of CD33+ myeloid-derived suppressor cells	39
		and poor prognosis of ovarian cancer patients	
	proteasomal degradation can be induced by	APC, UV-light, or cisplatin	40
	may mediate	the immune suppressive nature of KLGR1+ T cells	41
	binds to BRCA1 promoter in ovarian cancer cell lines,	repressing transcription and affecting sensitivity to PARP inhibitors	42
	overexpression reduces sensitivity to cisplatin	in non-small cell lung and esophageal squamous cell carcinoma	43, 44
RAD51B	variant rs2588809	is associated with breast cancer risk	21, 45
		is linked with the survival variant rs78150318 with R2=0.0016, D'=0.4677	
	silencing is associated with	increased genomic instability	46, 47
	somatic alterations in BRCA1-defective breast cancer	are associated with poor overall survival of patients	48
CREB5	is repressed by miR-29c	to inhibit proliferation in a preneoplastic triple-negative breast cancer model	49
	high expression in ER-positive mammary tumors	is associated with good prognosis with HR = 0.61 [0.50 - 0.74], P = 5.7E-7 (FDR 1%)	17
ASPH	locus is amplified in a subgroup of primary tumors		50
	becomes upregulated in response to	IGF1 exposure via MAPK and PI3K/Akt pathways	51
	expression is inversely correlated with	tamoxifen sensitivity of breast cancer cell lines	
	high expression predicts poor outcome	from endocrine therapy for patients with luminal B breast cancer	
	proteins and peptides are highly immunogenic	in induced dendritic cells of hepatocellular carcinoma patients	52, 53
		and may thus be a target for immunotherapy in hepatocellular carcinoma	
GATA3	is a transcription factor		54-56
	regulates differentiation of	mammary gland luminal cells	54, 55, 57
	silencing in mammary luminal cells	blocks differentiation	
		increased proliferation	
		leads to abnormalities nuclear size and orientation	
		causes cell detachment and increased cell death	
	knock-down in mice causes severe defects	in mammary gland formation and lactation	54, 55
	modulates ESR1 binding profile	by enabling ESR1 access to chromatin of limited active histone composition	56
	high expression in mammary tumors	is associated with hormone-positive/luminal tumor phenotype and low grade	58-62
		is associated with good patient prognosis	58, 59, 63-65
		does not affect prognosis	60, 62
		is associated with shorter locoregional relapse time in premenopausal patients	66
		is associated with decreased rate of pathologic complete response to neoadjuvant chemotherapy	61
		is associated with increased probability of GATA3 somatic mutation	65
	regulates spindle orientation	in prostate epithelial cells	67
	has diverse roles	in differentiation and homeostasis of immune cells	68

Gene	Function		Ref.
ZNF644	is a component of G9a/GLP complex	which represses transcription via H3K9 methylation	69, 70
		which interacts with polycomb repressor complex	
		which is a part of the replisome, required to prevent replication-associated DNA damage	
	recognizes the DNA target sequence		69
	silencing in vitro	decreased proliferation	71
		sensitized to replication stress	
		increased DNA damage in replicating cells	
	high expression in mammary tumors	is associated with poor prognosis with HR = 1.43 [1.21 - 1.69], P = 2.2E-5 (FDR 2%)	17
CLASP1	regulates	microtubule dynamics during mitosis	72, 73
	localizes	to the outer region of kinetochore (korona)	
		near the kinetochore-attached microtubule plus-ends	
	knock-down in human and murine cells	leads to chromosomal instability	73
	is required for	invasion through 3D matrix	74
		enhancing compression- resistance of growing microtubules	
	high expression in mammary tumors	is associated with good prognosis with HR = 0.71 [0.63 - 0.80], P = 4.9E-9 (FDR 1%)	17
NIFK	nucleolar protein interacting with the FHA domain	of MKI67	75
	overexpression in ling cancer cell lines	enhances proliferation, migration, and invasion	
	regulates TCF4/β-catenin	via repression of RUNX1 and CK1α	
	high expression in mammary tumors	is associated with poor patient survival	
	is phosphorylated by	CDK1 and GSK3	76
	transcription is induced by	c-Myc and estrogen	
	knock-down in osteosarcoma cell line	induced cell cycle arrest in G1 as a result of ribosomal stress	
	is required for ribosomal RNA maturation	especially in ITS1 (internal transcribed spacer 1) processing	
NIFK-AS1	suppresses macrophage M2 polarization;	M2 polarized macrophages participate in angiogenesis and promote tumor invasiveness	77
	overexpression in macrophages	suppressed the proliferation of estrogen-stimulated endometrial cancer cells	
	high expression in mammary tumors	is associated with good prognosis with HR = 0.64 [0.54 - 0.74], P = 1.5E-8 (FDR 1%)	17
TFCP2L1	is required for pluripotency	of embryonic stem cells	78
	together with Zf5, Ctcf, E2f1, and Myc	predicts the targets of polycomb repressor complex	79
	low expression in mammary tumors	is associated with poor prognosis with HR = 1.41 [1.20 - 1.67], P = 3.1E-5 (FDR 5%)	17
ARHGEF39	overexpression in gastric and non-small cell lung	cancer cell lines induces proliferation, migration, and invasion	80, 81
		increases Akt phophorylation activating the Akt/PI3K pathway	
		increases P38 and ATF2 phophorylation and activates MAPK pathway	
		increases the cellular levels of Cyclin A2, Cyclin D1, and MMP2	
		increases Rac1 activation	
	knockdown in gastric and non-small cell lung	cancer cell lines represses proliferation, migration, and invasion	
TPM2	binds actin	to stabilize microfilaments	82
	in involved in	cytokinesis, cellular vesicle transport, proliferation, migration, and apoptosis	
	expression is reduced	in breast cancer in comparison to adjacent normal tissue	
		in hypoxic conditions	
	silencing in breast cancer cell lines	increases invasion and migration	
		contributes to paclitaxel resistance	
	silencing in HeLa and U2OS cells	induces lysosomal destabilization and lysosomal cell death	83
		sensitizes to cisplatin, siramesine, and etoposide	
	low protein expression in mammary tumors	is associated with poor patient survival	82
	low mRNA expression in mammary tumors	is associated with good survival with HR = 0.64 [0.57 - 0.72], P = 2.9E-14 (FDR 1%)	17

Gene	Function		Ref.
GBA2	Bile acid b-glucosidase	breaks down glucocylceramide (outside lysosomes)	84
	Multi-drug resistant MCF-7	has high levels of glucocylceramide due to high glucocylceramide synthase activity	
	Glucocylceramide synthase repression	increases drug sensitivity in multi-drug resistant MCF-7	
	Overexpression of GBA2	DOES NOT have the same effect in multi-drug resistant MCF-7	
	low expression in mammary tumors	is associated with poor survival with HR = 1.58 [1.35 - 1.85], P = 5.6E-9 (FDR 1%)	17
	silencing leads to accumulation of glucosylceramide	in plasma membrane, affecting actin and microtubule dynamics	85
RUSC2	silencing in lung cancer cell lines	reduced directional migration (chemotaxis)	86
		did not affect random migration (chemokinesis)	
		causes defective Golgi orientation for chemotaxis	
	is required for EGFR-induced	GIT2 (G protein-coupled receptor kinase interacting ArfGAP 2) phosphorylation	
		directional cell migration	
CD72	interaction with CD5 is required for	regulatory B cell and regulatory T cell reciprocal stimulation	87
	is a membrane-bound receptor for CD100	in T cells	88
	interaction with CD100	is required for T cell activation	
TRAV	T cell receptor alpha variable region genes		

Supplementary Table 7. Survival associations in the Breast Cancer Association Consortium (BCAC) data.

Variant	BCAC: analysis group	HR [95% CI]	CIMBA: analysis group	HR [95% CI]	CIMBA: analysis group 2	HR [95% CI]
rs12126206	ER-	0.95 [0.83-1.08]	BRCA1 all BC	1.86 [1.50 - 2.32]		
rs5028286	ER-	1.09 [0.99-1.21]	BRCA1 all BC	1.74 [1.42 - 2.12]		
rs736418	ER-	1.01 [0.93-1.09]	BRCA1 all BC	0.60 [0.49 - 0.73]		
rs147857072	ER-	1.00 [0.67-1.49]	BRCA1 all BC	3.41 [2.32 - 5.00]		
rs59010985	ER-	0.96 [0.87-1.06]	BRCA1 all BC	0.58 [0.47 - 0.72]		
rs537497819			BRCA1 all BC	1.49 [1.27 - 1.74]		
rs1829314	ER-	1.10 [0.92-1.31]	BRCA1 ER-	0 [0 - 0]		
rs57025206	ER-	1.06 [0.86-1.29]	BRCA1 ER-	4.37 [3.03 - 6.30]		
rs11245414	ER-	1.00 [0.93-1.08]	BRCA1 ER-	0.56 [0.46 - 0.70]		
rs78150318	ER-	0.94 [0.78-1.15]	BRCA1 ER-	0 [0 - 0]		
rs551383190			BRCA1/BRCA2	0.61 [0.51 - 0.73]		
rs117422049	All	1.15 [0.98-1.35]	BRCA1/BRCA2	2.80 [1.89 - 4.13]		
rs4879914	All	0.99 [0.96-1.02]	BRCA1/BRCA2	1.30 [1.17 - 1.44]		
rs2320070	All	0.99 [0.95-1.03]	BRCA1/BRCA2	1.39 [1.22 - 1.58]		
rs372812554			BRCA2 under 40y	1.75 [1.33 - 2.31]	BRCA2 over 40y	0.75 [0.59 - 0.96]
rs2109815	ER+	1.03 [0.98-1.08]	BRCA2 under 40y	1.65 [1.24 - 2.19]	BRCA2 over 40y	0.75 [0.59 - 0.95]
rs35431863	ER+	1.01 [0.97-1.06]	BRCA2 under 40y	0.55 [0.43 - 0.71]	BRCA2 over 40y	1.32 [1.09 - 1.60]
rs11255420	ER+	0.98 [0.93-1.03]	BRCA2 under 40y	0.59 [0.41 - 0.83]	BRCA2 over 40y	1.55 [1.24 - 1.93]

Supplementary References

1. Gu S, Liang H, Qi D, Mao L, Mao G, Qian L, Zhang S. Knockdown of KIF26B inhibits breast cancer cell proliferation, migration, and invasion. Onco Targets Ther 11:3195-3203, 2018.

2. Teng Y, Guo B, Mu X, Liu S. KIF26B promotes cell proliferation and migration through the FGF2/ERK signaling pathway in breast cancer. Biomed Pharmacother 108:766-773, 2018.

3. Wang Q, Zhao ZB, Wang G, Hui Z, Wang MH, Pan JF, Zheng H. High expression of KIF26B in breast cancer associates with poor prognosis. PLoS One 8:e61640, 2013.

4. Guillabert-Gourgues A, Jaspard-Vinassa B, Bats M, Sewduth RN, Franzl N, Peghaire C, Jeanningros S, Moreau C, Roux E, Larrieu-Lahargue F, Dufourcq P, Couffinhal T, Duplàa C. Kif26b controls endothelial cell polarity through the dishevelled/Daam1-dependent planar cell polarity-signaling pathway. Mol Biol Cell 27:941-953, 2016.

5. Pu Y, Yi Q, Zhao F, Wang H, Cai W, Cai S. MiR-20a-5p represses multi-drug resistance in osteosarcoma by targeting the KIF26B gene. Cancer Cell Int 16:64, 2016.

6. Chi C, Murphy LC, Hu P. Recurrent copy number alterations in young women with breast cancer. Oncotarget 9:11541-11558, 2018.

7. Curtis C, Shah SP, Chin SF, Turashvili G, Rueda OM, Dunning MJ, Speed D, Lynch AG, Samarajiwa S, Yuan Y, Graf S, Ha G, Haffari G, Bashashati A, Russell R, McKinney S, METABRIC Group, Langerod A, Green A, Provenzano E, Wishart G, Pinder S, Watson P, Markowetz F, Murphy L, Ellis I, Purushotham A, Borresen-Dale AL, Brenton JD, Tavare S, Caldas C, Aparicio S. The genomic and transcriptomic architecture of 2,000 breast tumours reveals novel subgroups. Nature 486:346-352, 2012.

8. Yin M, Lu M, Yao G, Tian H, Lian J, Liu L, Liang M, Wang Y, Sun F. Transactivation of microRNA-383 by steroidogenic factor-1 promotes estradiol release from mouse ovarian granulosa cells by targeting RBMS1. Mol Endocrinol 26:1129-1143, 2012.

9. Jiang J, Xie C, Liu Y, Shi Q, Chen Y. Up-regulation of miR-383-5p suppresses proliferation and enhances chemosensitivity in ovarian cancer cells by targeting TRIM27. Biomed Pharmacother 109:595-601, 2019.

10. Wang Z, Zhou Y, Hu X, Chen W, Lin X, Sun L, Xu X, Hong W, Wang T. RILP suppresses invasion of breast cancer cells by modulating the activity of RalA through interaction with RalGDS. Cell Death Dis 6:e1923, 2015.

11. Ward Y, Wang W, Woodhouse E, Linnoila I, Liotta L, Kelly K. Signal pathways which promote invasion and metastasis: Critical and distinct contributions of extracellular signal-regulated kinase and ral-specific guanine exchange factor pathways. Mol Cell Biol 21:5958-5969, 2001.

12. Bhattacharya M, Anborgh PH, Babwah AV, Dale LB, Dobransky T, Benovic JL, Feldman RD, Verdi JM, Rylett RJ, Ferguson SSG. Beta-arrestins regulate a ral-GDS ral effector pathway that mediates cytoskeletal reorganization. Nat Cell Biol 4:547-555, 2002.

13. Lee YH, Kim JH, Song GG. Genome-wide pathway analysis of breast cancer. Tumour Biol 35:7699-7705, 2014.

14. Conway K, Edmiston SN, May R, Kuan PF, Chu H, Bryant C, Tse CK, Swift-Scanlan T, Geradts J, Troester MA, Millikan RC. DNA methylation profiling in the carolina breast cancer study defines cancer subclasses differing in clinicopathologic characteristics and survival. Breast Cancer Res 16:45-6, 2014.

15. Ronneberg JA, Fleischer T, Solvang HK, Nordgard SH, Edvardsen H, Potapenko I, Nebdal D, Daviaud C, Gut I, Bukholm I, Naume B, Borresen-Dale AL, Tost J, Kristensen V. Methylation profiling with a panel of cancer related genes: Association with estrogen receptor, TP53 mutation status and expression subtypes in sporadic breast cancer. Mol Oncol 5:61-76, 2011.

16. Chang JW, Kuo WH, Lin CM, Chen WL, Chan SH, Chiu MF, Chang IS, Jiang SS, Tsai FY, Chen CH, Huang PH, Chang KJ, Lin KT, Lin SC, Wang MY, Uen YH, Tu CW, Hou MF, Tsai SF, Shen CY, Tung SL, Wang LH. Wild-type p53 upregulates an early onset breast cancer-associated gene GAS7 to suppress metastasis via GAS7-CYFIP1-mediated signaling pathway. Oncogene 37:4137-4150, 2018.

17. Gyorffy B, Lanczky A, Eklund AC, Denkert C, Budczies J, Li Q, Szallasi Z. An online survival analysis tool to rapidly assess the effect of 22,277 genes on breast cancer prognosis using microarray data of 1,809 patients. Breast Cancer Res Treat 123:725-731, 2010.

18. Hung F, Chao CC-. Knockdown of growth-arrest-specific gene 7b (gas7b) using short-hairpin RNA desensitizes neuroblastoma cells to cisplatin: Implications for preventing apoptosis of neurons. J Neurosci Res 88:3578-3587, 2010.

19. Li D, Zhang B, Hu C. Oxaliplatin inhibits proliferation and migration of human hepatocellular carcinoma cells via GAS7C and the N-WASP/FAK/F-actin pathway. Acta Biochim Biophys Sin (Shanghai) 49:581-587, 2017.

20. Ping W, Gao Y, Fan X, Li W, Deng Y, Fu X. MiR-181a contributes gefitinib resistance in non-small cell lung cancer cells by targeting GAS7. Biochem Biophys Res Commun 495:2482-2489, 2018.

21. Michailidou K, Hall P, Gonzalez-Neira A, Ghoussaini M, Dennis J, Milne RL, Schmidt MK, Chang-Claude J, Bojesen SE, Bolla MK, Wang Q, Dicks E, Lee A, Turnbull C, Rahman N, Breast and Ovarian Cancer Susceptibility Collaboration, Fletcher O, Peto J, Gibson L, Dos Santos Silva I, Nevanlinna H, Muranen TA, Aittomaki K, Blomqvist C, Czene K, Irwanto A, Liu J, Waisfisz Q, Meijers-Heijboer H, Adank M, Hereditary Breast and Ovarian Cancer Research Group Netherlands, (HEBON), van der Luijt, R. B., Hein R, Dahmen N, Beckman L, Meindl A, Schmutzler RK, Muller-Myhsok B, Lichtner P, Hopper JL, Southey MC, Makalic E, Schmidt DF, Uitterlinden AG, Hofman A, Hunter DJ, Chanock SJ, Vincent D, Bacot F, Tessier DC, Canisius S, Wessels LF, Haiman CA, Shah M, Luben R, Brown J, Luccarini C, Schoof N, Humphreys K, Li J, Nordestgaard BG, Nielsen SF, Flyger H, Couch FJ, Wang X, Vachon C, Stevens KN, Lambrechts D, Moisse M, Paridaens R, Christiaens MR, Rudolph A, Nickels S, Flesch-Janys D, Johnson N, Aitken Z, Aaltonen K, Heikkinen T, Broeks A, Veer LJ, van der Schoot, C. E., Guenel P, Truong T, Laurent-Puig P, Menegaux F, Marme F, Schneeweiss A, Sohn C, Burwinkel B, Zamora MP, Perez JI, Pita G, Alonso MR, Cox A, Brock IW, Cross SS, Reed MW, Sawyer EJ, Tomlinson I, Kerin MJ, Miller N, Henderson BE, Schumacher F, Le Marchand L, Andrulis IL, Knight JA, Glendon G, Mulligan AM, kConFab Investigators, stralian Ovarian Cancer Study Group, Lindblom A, Margolin S, Hooning MJ, Hollestelle A, van den Ouweland, A. M., Jager A, Bui QM, Stone J, Dite GS, Apicella C, Tsimiklis H, Giles GG, Severi G, Baglietto L, Fasching PA, Haeberle L, Ekici AB, Beckmann MW, Brenner H, Muller H, Arndt V, Stegmaier C, Swerdlow A, Ashworth A, Orr N, Jones M, Figueroa J, Lissowska

J, Brinton L, Goldberg MS, Labreche F, Dumont M, Winqvist R, Pylkas K, Jukkola-Vuorinen A, Grip M, Brauch H, Hamann U, Bruning T, GENICA (Gene Environment Interaction and Breast Cancer in Germany) Network, Radice P, Peterlongo P, Manoukian S, Bonanni B, Devilee P, Tollenaar RA, Seynaeve C, van Asperen CJ, Jakubowska A, Lubinski J, Jaworska K, Durda K, Mannermaa A, Kataja V, Kosma VM, Hartikainen JM, Bogdanova NV, Antonenkova NN, Dork T, Kristensen VN, Anton-Culver H, Slager S, Toland AE, Edge S, Fostira F, Kang D, Yoo KY, Noh DY, Matsuo K, Ito H, Iwata H, Sueta A, Wu AH, Tseng CC, Van Den Berg D, Stram DO, Shu XO, Lu W, Gao YT, Cai H, Teo SH, Yip CH, Phuah SY, Cornes BK, Hartman M, Miao H, Lim WY, Sng JH, Muir K, Lophatananon A, Stewart-Brown S, Siriwanarangsan P, Shen CY, Hsiung CN, Wu PE, Ding SL, Sangrajrang S, Gaborieau V, Brennan P, McKay J, Blot WJ, Signorello LB, Cai Q, Zheng W, Deming-Halverson S, Shrubsole M, Long J, Simard J, Garcia-Closas M, Pharoah PD, Chenevix-Trench G, Dunning AM, Benitez J, Easton DF. Large-scale genotyping identifies 41 new loci associated with breast cancer risk. Nat Genet 45:353-361, 2013.

22. Yuan J, Zhang N, Zhu H, Liu J, Xing H, Ma F, Yang M. CHST9 rs1436904 genetic variant contributes to prognosis of triple-negative breast cancer. Sci Rep 7:1180-6, 2017.

23. Schabla NM, Mondal K, Swanson PC. DCAF1 (VprBP): Emerging physiological roles for a unique dual-service E3 ubiquitin ligase substrate receptor. J Mol Cell Biol , 2018.

24. Britschgi A, Duss S, Kim S, Couto JP, Brinkhaus H, Koren S, De Silva D, Mertz KD, Kaup D, Varga Z, Voshol H, Vissieres A, Leroy C, Roloff T, Stadler MB, Scheel CH, Miraglia LJ, Orth AP, Bonamy GMC, Reddy VA, Bentires-Alj M. The hippo kinases LATS1 and 2 control human breast cell fate via crosstalk with ERα. Nature 541:541-545, 2017.

25. Lee JM, Lee JS, Kim H, Kim K, Park H, Kim J, Lee SH, Kim IS, Kim J, Lee M, Chung CH, Seo S, Yoon J, Ko E, Noh D, Kim KI, Kim KK, Baek SH. EZH2 generates a methyl degron that is recognized by the DCAF1/DDB1/CUL4 E3 ubiquitin ligase complex. Mol Cell 48:572-586, 2012.

26. Guo Z, Kong Q, Liu C, Zhang S, Zou L, Yan F, Whitmire JK, Xiong Y, Chen X, Wan YY. DCAF1 controls T-cell function via p53-dependent and -independent mechanisms. Nat Commun 7:10307, 2016.

27. Hossain D, Ferreira Barbosa JA, Cohen ÉA, Tsang WY. HIV-1 vpr hijacks EDD-DYRK2-DDB1DCAF1 to disrupt centrosome homeostasis. J Biol Chem 293:9448-9460, 2018.

28. Zhang W, Wu M, Chong Q, Zhang M, Zhang X, Hu L, Zhong Y, Qian P, Kong X, Tan S, Li G, Ding K, Lobie PE, Zhu T. Loss of estrogen-regulated MIR135A1 at 3p21.1 promotes tamoxifen resistance in breast cancer. Cancer Res 78:4915-4928, 2018.

29. Yan L, Chen Z, Li-Li n, Chen J, Wei W, Mo X, Qin Y, Lin Y, Chen J. miR-135a promotes gastric cancer progression and resistance to oxaliplatin. Oncotarget 7:70699-70714, 2016.

30. Zhang T, Wang N. miR-135a confers resistance to gefitinib in non-small cell lung cancer cells by upregulation of RAC1. Oncol Res 26:1191-1200, 2018.

31. Maitra A, Wistuba II, Washington C, Virmani AK, Ashfaq R, Milchgrub S, Gazdar AF, Minna JD. High-resolution chromosome 3p allelotyping of breast carcinomas and precursor lesions demonstrates frequent loss of heterozygosity and a discontinuous pattern of allele loss. Am J Pathol 159:119-130, 2001.

32. Martinez A, Walker RA, Shaw JA, Dearing SJ, Maher ER, Latif F. Chromosome 3p allele loss in early invasive breast cancer: Detailed mapping and association with clinicopathological features. MP, Mol Pathol 54:300-306, 2001.

33. Zhang P, Xiao Z, Wang S, Zhang M, Wei Y, Hang Q, Kim J, Yao F, Rodriguez-Aguayo C, Ton BN, Lee M, Wang Y, Zhou Z, Zeng L, Hu X, Lawhon SE, Siverly AN, Su X, Li J, Xie X, Cheng X, Liu L, Chang H, Chiang S, Lopez-Berestein G, Sood AK, Chen J, You MJ, Sun S, Liang H, Huang Y, Yang X, Sun D, Sun Y, Hung M, Ma L. ZRANB1 is an EZH2 deubiquitinase and a potential therapeutic target in breast cancer. Cell Rep 23:823-837, 2018.

34. Bai SW, Herrera-Abreu MT, Rohn JL, Racine V, Tajadura V, Suryavanshi N, Bechtel S, Wiemann S, Baum B, Ridley AJ. Identification and characterization of a set of conserved and new regulators of cytoskeletal organization, cell morphology and migration. BMC Biol 9:54, 2011.

35. Jin J, Xie X, Xiao Y, Hu H, Zou Q, Cheng X, Sun S. Epigenetic regulation of the expression of Il12 and Il23 and autoimmune inflammation by the deubiquitinase trabid. Nat Immunol 17:259-268, 2016.

36. Bergman LM, Birts CN, Darley M, Gabrielli B, Blaydes JP. CtBPs promote cell survival through the maintenance of mitotic fidelity. Mol Cell Biol 29:4539-4551, 2009.

37. Kim TW, Kang B, Jang H, Kwak S, Shin J, Kim H, Lee S, Lee S, Lee J, Kim J, Kim S, Cho E, Kim JH, Park KS, Che J, Han DW, Kang MJ, Yi EC, Youn H. Ctbp2 modulates NuRD-mediated deacetylation of H3K27 and facilitates PRC2-mediated H3K27me3 in active embryonic stem cell genes during exit from pluripotency. Stem Cells 33:2442-2455, 2015.

38. Yang X, Sun Y, Li H, Shao Y, Zhao D, Yu W, Fu J. C-terminal binding protein-2 promotes cell proliferation and migration in breast cancer via suppression of p16INK4A. Oncotarget 8:26154-26168, 2017.

39. Cui TX, Kryczek I, Zhao L, Zhao E, Kuick R, Roh MH, Vatan L, Szeliga W, Mao Y, Thomas DG, Kotarski J, Tarkowski R, Wicha M, Cho K, Giordano T, Liu R, Zou W. Myeloid-derived suppressor cells enhance stemness of cancer cells by inducing microRNA101 and suppressing the corepressor CtBP2. Immunity 39:611-621, 2013.

40. Birts CN, Harding R, Soosaipillai G, Halder T, Azim-Araghi A, Darley M, Cutress RI, Bateman AC, Blaydes JP. Expression of CtBP family protein isoforms in breast cancer and their role in chemoresistance. Biol Cell 103:1-19, 2010.

41. Li L, Wan S, Tao K, Wang G, Zhao E. KLRG1 restricts memory T cell antitumor immunity. Oncotarget 7:61670-61678, 2016.

42. May T, Yang J, Shoni M, Liu S, He H, Gali R, Ng S, Crum C, Berkowitz RS, Ng S. BRCA1 expression is epigenetically repressed in sporadic ovarian cancer cells by overexpression of C-terminal binding protein 2. Neoplasia 15:600-608, 2013.

43. Wang DP, Gu LL, Xue Q, Chen H, Mao GX. CtBP2 promotes proliferation and reduces drug sensitivity in non-small cell lung cancer via the wnt/β-catenin pathway. Neoplasma 65:888-897, 2018.

44. Shi H, Mao Y, Ju Q, Wu Y, Bai W, Wang P, Zhang Y, Jiang M. C-terminal binding protein 2 mediates cisplatin chemoresistance in esophageal cancer cells via the inhibition of apoptosis. Int J Oncol 53:167-176, 2018.

45. Thomas G, Jacobs KB, Kraft P, Yeager M, Wacholder S, Cox DG, Hankinson SE, Hutchinson A, Wang Z, Yu K, Chatterjee N, Garcia-Closas M, Gonzalez-Bosquet J, Prokunina-Olsson L, Orr N, Willett WC, Colditz GA, Ziegler RG, Berg CD, Buys SS, McCarty CA, Feigelson HS, Calle EE, Thun MJ, Diver R, Prentice R, Jackson R, Kooperberg C, Chlebowski R, Lissowska J, Peplonska B, Brinton LA, Sigurdson A, Doody M, Bhatti P, Alexander BH, Buring J, Lee IM, Vatten LJ, Hveem K, Kumle M, Hayes RB, Tucker M, Gerhard DS, Fraumeni JF,Jr, Hoover RN, Chanock SJ, Hunter DJ. A multistage genome-wide association study in breast cancer identifies two new risk alleles at 1p11.2 and 14q24.1 (RAD51L1). Nat Genet 41:579-584, 2009.

46. Suwaki N, Klare K, Tarsounas M. RAD51 paralogs: Roles in DNA damage signalling, recombinational repair and tumorigenesis. Semin Cell Dev Biol 22:898-905, 2011.

47. Zhu B, Mukherjee A, Machiela MJ, Song L, Hua X, Shi J, Garcia-Closas M, Chanock SJ, Chatterjee N. An investigation of the association of genetic susceptibility risk with somatic mutation burden in breast cancer. Br J Cancer 115:752-760, 2016.

48. Takada M, Nagai S, Haruta M, Sugino RP, Tozuka K, Takei H, Ohkubo F, Inoue K, Kurosumi M, Miyazaki M, Sato-Otsubo A, Sato Y, Ogawa S, Kaneko Y. BRCA1 alterations with additional defects in DNA damage response genes may confer chemoresistance to BRCA-like breast cancers treated with neoadjuvant chemotherapy. Genes Chromosomes Cancer 56:405-420, 2017.

49. Bhardwaj A, Singh H, Rajapakshe K, Tachibana K, Ganesan N, Pan Y, Gunaratne PH, Coarfa C, Bedrosian I. Regulation of miRNA-29c and its downstream pathways in preneoplastic progression of triple-negative breast cancer. Oncotarget 8:19645-19660, 2017.

50. Kadota M, Sato M, Duncan B, Ooshima A, Yang HH, Diaz-Meyer N, Gere S, Kageyama S, Fukuoka J, Nagata T, Tsukada K, Dunn BK, Wakefield LM, Lee MP. Identification of novel gene amplifications in breast cancer and coexistence of gene amplification with an activating mutation of PIK3CA. Cancer Res 69:7357-7365, 2009.

51. Shimoda M, Hori A, Wands JR, Tsunashima R, Naoi Y, Miyake T, Tanei T, Kagara N, Shimazu K, Kim SJ, Noguchi S. Endocrine sensitivity of estrogen receptor-positive breast cancer is negatively correlated with aspartate-β-hydroxylase expression. Cancer Sci 108:2454-2461, 2017.

52. Tomimaru Y, Mishra S, Safran H, Charpentier KP, Martin W, De Groot AS, Gregory SH, Wands JR. Aspartate-β-hydroxylase induces epitope-specific T cell responses in hepatocellular carcinoma. Vaccine 33:1256-1266, 2015.

53. Shimoda M, Tomimaru Y, Charpentier KP, Safran H, Carlson RI, Wands J. Tumor progression-related transmembrane protein aspartate-β-hydroxylase is a target for immunotherapy of hepatocellular carcinoma. J Hepatol 56:1129-1135, 2012.

54. Kouros-Mehr H, Slorach EM, Sternlicht MD, Werb Z. GATA-3 maintains the differentiation of the luminal cell fate in the mammary gland. Cell 127:1041-1055, 2006.

55. Asselin-Labat M, Sutherland KD, Barker H, Thomas R, Shackleton M, Forrest NC, Hartley L, Robb L, Grosveld FG, van der Wees J, Lindeman GJ, Visvader JE. Gata-3 is an essential regulator of mammary-gland morphogenesis and luminal-cell differentiation. Nat Cell Biol 9:201-209, 2007.

56. Theodorou V, Stark R, Menon S, Carroll JS. GATA3 acts upstream of FOXA1 in mediating ESR1 binding by shaping enhancer accessibility. Genome Res 23:12-22, 2013.

57. Shahi P, Wang C-, Chou J, Hagerling C, Gonzalez Velozo H, Ruderisch A, Yu Y, Lai M-, Werb Z. GATA3 targets semaphorin 3B in mammary epithelial cells to suppress breast cancer progression and metastasis. Oncogene 36:5567-5575, 2017.

58. Mehra R, Varambally S, Ding L, Shen R, Sabel MS, Ghosh D, Chinnaiyan AM, Kleer CG. Identification of GATA3 as a breast cancer prognostic marker by global gene expression meta-analysis. Cancer Res 65:11259-11264, 2005.

59. Yoon, Nam K., BS|Maresh, Erin L., BS|Shen, Dejun, MD|Elshimali, Yahya, MD|Apple, Sophia, MD|Horvath, Steve, PhD|Mah, Vei, MD|Bose, Shikha, MD|Chia, David, PhD|Chang, Helena R., MD|Goodglick, Lee, PhD. Higher levels of GATA3 predict better survival in women with breast cancer. Human Pathology 41:1794-1801, 2010.

60. Albergaria A, Paredes J, Sousa B, Milanezi F, Carneiro V, Bastos J, Costa S, Vieira D, Lopes N, Lam EW, Lunet N, Schmitt F. Expression of FOXA1 and GATA-3 in breast cancer: The prognostic significance in hormone receptor-negative tumours. Breast Cancer Res 11:R40, 2009.

61. Tominaga N, Naoi Y, Shimazu K, Nakayama T, Maruyama N, Shimomura A, Kim SJ, Tamaki Y, Noguchi S. Clinicopathological analysis of GATA3-positive breast cancers with special reference to response to neoadjuvant chemotherapy. Ann Oncol 23:3051-3057, 2012.

62. Gulbahce, H. Evin, MD|Sweeney, Carol, PhD|Surowiecka, Maria, MD|Knapp, Dennis, BS|Varghese, Linda, MD|Blair, Cindy K., PhD. Significance of GATA-3 expression in outcomes of patients with breast cancer who received systemic chemotherapy and/or hormonal therapy and clinicopathologic features of GATA-3–positive tumors. Human Pathology 44:2427-2431, 2013.

63. Ciocca, Vincenzo, DO|Daskalakis, Constantine, ScD|Ciocca, Robin M., DO|Ruiz-Orrico, Alejandra, MD|Palazzo, Juan P., MD. The significance of GATA3 expression in breast cancer: A 10-year follow-up study. Human Pathology 40:489-495, 2009.

64. Jenssen T-, Kuo WP, Stokke T, Hovig E. Associations between gene expressions in breast cancer and patient survival. Hum Genet 111:411-420, 2002.

65. Liu J, Prager-van der Smissen, Wendy J. C., Look MP, Sieuwerts AM, Smid M, Meijer-van Gelder ME, Foekens JA, Hollestelle A, Martens JWM. GATA3 mRNA expression, but not mutation, associates with longer progression-free survival in ER-positive breast cancer patients treated with first-line tamoxifen for recurrent disease. Cancer Lett 376:104-109, 2016.

66. Bollet MA, Savignoni A, De Koning L, Tran-Perennou C, Barbaroux C, Degeorges A, Sigal-Zafrani B, Almouzni G, Cottu P, Salmon R, Servant N, Fourquet A, de Cremoux P. Tumor aromatase expression as a

prognostic factor for local control in young breast cancer patients after breast-conserving treatment. Breast Cancer Res 11:R54, 2009.

67. Shafer MER, Nguyen AHT, Tremblay M, Viala S, Béland M, Bertos NR, Park M, Bouchard M. Lineage specification from prostate progenitor cells requires Gata3-dependent mitotic spindle orientation. Stem Cell Reports 8:1018-1031, 2017.

68. Wan YY. GATA3: A master of many trades in immune regulation. Trends Immunol 35:233-242, 2014.

69. Bian C, Chen Q, Yu X. The zinc finger proteins ZNF644 and WIZ regulate the G9a/GLP complex for gene repression. Elife 4, 2015.

70. Mozzetta C, Pontis J, Fritsch L, Robin P, Portoso M, Proux C, Margueron R, Ait-Si-Ali S. The histone H3 lysine 9 methyltransferases G9a and GLP regulate polycomb repressive complex 2-mediated gene silencing. Mol Cell 53:277-289, 2014.

71. Dungrawala H, Rose KL, Bhat KP, Mohni KN, Glick GG, Couch FB, Cortez D. The replication checkpoint prevents two types of fork collapse without regulating replisome stability. Mol Cell 59:998-1010, 2015.

72. Maiato H, Fairley EAL, Rieder CL, Swedlow JR, Sunkel CE, Earnshaw WC. Human CLASP1 is an outer kinetochore component that regulates spindle microtubule dynamics. Cell 113:891-904, 2003.

73. Pereira AL, Pereira AJ, Maia ARR, Drabek K, Sayas CL, Hergert PJ, Lince-Faria M, Matos I, Duque C, Stepanova T, Rieder CL, Earnshaw WC, Galjart N, Maiato H. Mammalian CLASP1 and CLASP2 cooperate to ensure mitotic fidelity by regulating spindle and kinetochore function. Mol Biol Cell 17:4526-4542, 2006.

74. Bouchet BP, Noordstra I, van Amersfoort M, Katrukha EA, Ammon Y, Ter Hoeve ND, Hodgson L, Dogterom M, Derksen PWB, Akhmanova A. Mesenchymal cell invasion requires cooperative regulation of persistent microtubule growth by SLAIN2 and CLASP1. Dev Cell 39:708-723, 2016.

75. Lin T, Su C, Wu P, Lai T, Pan W, Jan Y, Chang Y, Yeh C, Chen C, Ger L, Chang H, Yang C, Huang M, Liu Y, Lin Y, Shyy JY-, Tsai M, Hsiao M. The nucleolar protein NIFK promotes cancer progression via CK1α/β-catenin in metastasis and ki-67-dependent cell proliferation. Elife 5, 2016.

76. Pan W, Tsai H, Wang S, Hsiao M, Wu P, Tsai M. The RNA recognition motif of NIFK is required for rRNA maturation during cell cycle progression. RNA Biol 12:255-267, 2015.

77. Zhou Y, Zhao W, Mao L, Wang Y, Xia L, Cao M, Shen J, Chen J. Long non-coding RNA NIFK-AS1 inhibits M2 polarization of macrophages in endometrial cancer through targeting miR-146a. Int J Biochem Cell Biol 104:25-33, 2018.

78. Kotarba G, Krzywinska E, Grabowska AI, Taracha A, Wilanowski T. TFCP2/TFCP2L1/UBP1 transcription factors in cancer. Cancer Lett 420:72-79, 2018.

79. Liu Y, Shao Z, Yuan G. Prediction of polycomb target genes in mouse embryonic stem cells. Genomics 96:17-26, 2010.

80. Wang H, Li M, Tao X, Qian Y, Chen L, Tao G. ARHGEF39 promotes gastric cancer cell proliferation and migration via akt signaling pathway. Mol Cell Biochem 440:33-42, 2018.

81. Zhou H, Cai L, Zhang X, Li A, Miao Y, Li Q, Qiu X, Wang E. ARHGEF39 promotes tumor progression via activation of Rac1/P38 MAPK/ATF2 signaling and predicts poor prognosis in non-small cell lung cancer patients. Lab Invest 98:670-681, 2018.

82. Zhang J, Zhang J, Xu S, Zhang X, Wang P, Wu H, Xia B, Zhang G, Lei B, Wan L, Zhang D, Pang D. Hypoxia-induced TPM2 methylation is associated with chemoresistance and poor prognosis in breast cancer. Cell Physiol Biochem 45:692-705, 2018.

83. Groth-Pedersen L, Aits S, Corcelle-Termeau E, Petersen NHT, Nylandsted J, Jäättelä M. Identification of cytoskeleton-associated proteins essential for lysosomal stability and survival of human cancer cells. PLoS ONE 7:e45381, 2012.

84. Astudillo L, Therville N, Colacios C, Ségui B, Andrieu-Abadie N, Levade T. Glucosylceramidases and malignancies in mammals. Biochimie 125:267-280, 2016.

85. Raju D, Schonauer S, Hamzeh H, Flynn KC, Bradke F, Vom Dorp K, Dörmann P, Yildiz Y, Trötschel C, Poetsch A, Breiden B, Sandhoff K, Körschen HG, Wachten D. Accumulation of glucosylceramide in the absence of the beta-glucosidase GBA2 alters cytoskeletal dynamics. PLoS Genet 11:e1005063, 2015.

86. Duan B, Cui J, Sun S, Zheng J, Zhang Y, Ye B, Chen Y, Deng W, Du J, Zhu Y, Chen Y, Gu L. EGF-stimulated activation of Rab35 regulates RUSC2-GIT2 complex formation to stabilize GIT2 during directional lung cancer cell migration. Cancer Lett 379:70-83, 2016.

87. Zheng M, Xing C, Xiao H, Ma N, Wang X, Han G, Chen G, Hou C, Shen B, Li Y, Wang R. Interaction of CD5 and CD72 is involved in regulatory T and B cell homeostasis. Immunol Invest 43:705-716, 2014.

88. Jiang X, Björkström NK, Melum E. Intact CD100-CD72 interaction necessary for TCR-induced T cell proliferation. Front Immunol 8:765, 2017.

Supplementary note

Acknowledgements

We thank all the individuals who took part in these studies and all the researchers, clinicians, technicians and administrative staff who have enabled this work to be carried out; Sue Healey, in particular taking on the task of mutation classification with the late Olga Sinilnikova; Maggie Angelakos, Judi Maskiell, Gillian Dite, Helen Tsimiklis; members and participants in the New York site of the Breast Cancer Family Registry; members and participants in the Ontario Familial Breast Cancer Registry; Vilius Rudaitis and Laimonas Griškevičius; Drs Janis Eglitis, Anna Krilova and Aivars Stengrevics; Yuan Chun Ding and Linda Steele for their work in participant enrollment and biospecimen and data management; Bent Ejlertsen and Anne-Marie Gerdes for the recruitment and genetic counseling of participants; Alicia Barroso, Rosario Alonso and Guillermo Pita; Manoukian Siranoush, Bernard Peissel, Cristina Zanzottera, Milena Mariani, Daniela Zaffaroni, Bernardo Bonanni, Monica Barile, Irene Feroce, Mariarosaria Calvello, Alessandra Viel, Riccardo Dolcetti, Laura Ottini, Giuseppe Giannini, Laura Papi, Gabriele Lorenzo Capone, Liliana Varesco, Viviana Gismondi, Maria Grazia Tibiletti, Daniela Furlan, Antonella Savarese, Aline Martayan, Stefania Tommasi, Brunella Pilato; the personnel of the Cogentech Cancer Genetic Test Laboratory, Milan, Italy. Ms. JoEllen Weaver and Dr. Betsy Bove; Marta Santamariña, Ana Blanco, Miguel Aguado, Uxía Esperón and Belinda Rodríguez; IFE - Leipzig Research Centre for Civilization Diseases (Markus Loeffler, Joachim Thiery, Matthias Nüchter, Ronny Baber); We thank all participants, clinicians, family doctors, researchers, and technicians for their contributions and commitment to the DKFZ study and the collaborating groups in Lahore, Pakistan (Muhammad U. Rashid, Noor Muhammad, Sidra Gull, Seerat Bajwa, Faiz Ali Khan, Humaira Naeemi, Saima Faisal, Asif Loya, Mohammed Aasim Yusuf) and Bogota, Colombia (Diana Torres, Ignacio Briceno, Fabian Gil). Genetic Modifiers of Cancer Risk in BRCA1/2 Mutation Carriers (GEMO) study is a study from the National Cancer Genetics Network UNICANCER Genetic Group, France. We wish to pay a tribute to Olga M. Sinilnikova, who with Dominique Stoppa-Lyonnet initiated and coordinated GEMO until she sadly passed away on the 30th June 2014. The team in Lyon (Olga Sinilnikova, Mélanie Léoné, Laure Barjhoux, Carole Verny-Pierre, Sylvie Mazoyer, Francesca Damiola, Valérie Sornin) managed the GEMO samples until the biological resource centre was transferred to Paris in December 2015 (Noura Mebirouk, Fabienne Lesueur, Dominique Stoppa-Lyonnet). We want to thank all the GEMO collaborating groups for their contribution to this study: Coordinating Centre, Service de Génétique, Institut Curie, Paris, France: Muriel Belotti, Ophélie Bertrand, Anne-Marie Birot, Bruno Buecher, Sandrine Caputo, Anaïs Dupré, Emmanuelle Fourme, Marion Gauthier-Villars, Lisa Golmard, Claude Houdayer, Marine Le Mentec, Virginie Moncoutier, Antoine de Pauw, Claire Saule, Dominique Stoppa-Lyonnet, and Inserm U900, Institut Curie, Paris, France: Fabienne Lesueur, Noura Mebirouk.Contributing Centres : Unité Mixte de Génétique Constitutionnelle des Cancers Fréquents, Hospices Civils de Lyon - Centre Léon Bérard, Lyon, France: Nadia Boutry-Kryza, Alain Calender, Sophie Giraud, Mélanie Léone. Institut Gustave Roussy, Villejuif, France: Brigitte Bressac-de-Paillerets, Olivier Caron, Marine Guillaud-Bataille. Centre Jean Perrin, Clermont–Ferrand, France: Yves-Jean Bignon, Nancy Uhrhammer. Centre Léon Bérard, Lyon, France: Valérie Bonadona, Christine Lasset. Centre François Baclesse, Caen, France: Pascaline Berthet, Laurent Castera, Dominique Vaur. Institut Paoli Calmettes, Marseille, France: Violaine Bourdon, Catherine Noguès, Tetsuro Noguchi, Cornel Popovici, Audrey Remenieras, Hagay Sobol. CHU Arnaud-de-Villeneuve, Montpellier, France: Isabelle Coupier, Pascal Pujol. Centre Oscar Lambret, Lille, France: Claude Adenis, Aurélie Dumont, Françoise Révillion. Centre Paul Strauss, Strasbourg, France: Danièle Muller. Institut Bergonié, Bordeaux, France: Emmanuelle Barouk-Simonet, Françoise Bonnet, Virginie Bubien, Michel Longy, Nicolas Sevenet, Institut Claudius Regaud, Toulouse, France: Laurence Gladieff, Rosine Guimbaud, Viviane Feillel, Christine Toulas. CHU Grenoble, France: Hélène Dreyfus, Christine Dominique Leroux, Magalie Peysselon, Rebischung. CHU Dijon, France: Amandine Baurand, Geoffrey Bertolone, Fanny Coron, Laurence Faivre, Caroline Jacquot, Sarab Lizard. CHU St-Etienne, France: Caroline Kientz, Marine Lebrun, Fabienne Prieur. Hôtel Dieu Centre Hospitalier, Chambéry, France: Sandra Fert Ferrer. Centre Antoine Lacassagne, Nice, France: Véronique Mari. CHU Limoges, France: Laurence Vénat-Bouvet. CHU Nantes, France: Stéphane Bézieau, Capucine Delnatte. CHU Bretonneau, Tours and Centre Hospitalier de Bourges France: Isabelle Mortemousque. Groupe Hospitalier Pitié-Salpétrière, Paris, France: Chrystelle Colas, Florence Coulet, Florent Soubrier, Mathilde Warcoin. CHU Vandoeuvre-les-Nancy, France: Myriam Bronner, Johanna Sokolowska. CHU Besançon, France: Marie-Agnès Collonge-Rame, Alexandre Damette. CHU Poitiers, Centre Hospitalier d'Angoulême and Centre Hospitalier de Niort, France: Paul Gesta. Centre Hospitalier de La Rochelle : Hakima Lallaoui. CHU Nîmes Carémeau, France : Jean Chiesa. CHI Poissy, France: Denise Molina-Gomes. CHU Angers, France : Olivier Ingster; Ilse Coene en Brecht Crombez; Ilse Coene and Brecht Crombez; Alicia Tosar and Paula Diaque; Irja Erkkilä and Virpi Palola; HEBON thanks the study participants and the registration teams of IKNL and PALGA for part of the data collection; Hong Kong Sanatorium and Hospital; the Hungarian Breast and Ovarian Cancer Study Group members (Janos Papp, Aniko Bozsik, Timea Pocza, Zoltan Matrai, Miklos Kasler, Judit Franko, Maria Balogh, Gabriella Domokos, Judit Ferenczi, Department of Molecular Genetics, National Institute of Oncology, Budapest, Hungary) and the clinicians and patients for their contributions to this study; the Oncogenetics Group (VHIO) and the High Risk and Cancer Prevention Unit of the University Hospital Vall d'Hebron, and the Cellex Foundation for providing research facilities and equipment; the ICO Hereditary Cancer Program team led by Dr. Gabriel Capella; the ICO Hereditary Cancer Program team led by Dr. Gabriel Capella; Dr Martine Dumont for sample management and skillful assistance; Ana Peixoto, Catarina Santos and Pedro Pinto; members of the Center of Molecular Diagnosis, Oncogenetics Department and Molecular Oncology Research Center of Barretos Cancer Hospital; all the kConFab research nurses and staff, the heads and staff of the Family Cancer Clinics, and the Clinical Follow Up Study (which has received funding from the NHMRC, the National Breast Cancer Foundation, Cancer Australia, and the National Institute of Health (USA)) for their contributions to this resource, and the many families who contribute to kConFab; the KOBRA Study Group; Csilla Szabo (National Human Genome Research Institute, National Institutes of Health, Bethesda, MD, USA); Lenka Foretova and Eva Machackova (Department of Cancer Epidemiology and Genetics, Masaryk Memorial Cancer Institute and MF MU, Brno, Czech Republic); and Michal Zikan, Petr Pohlreich and Zdenek Kleibl (Oncogynecologic Center and Department of Biochemistry and Experimental Oncology, First Faculty of Medicine, Charles University, Prague, Czech Republic); Anne Lincoln, Lauren Jacobs; the NICCC National Familial Cancer Consultation Service team led by Sara Dishon, the lab team led by Dr. Flavio Lejbkowicz, and the research field operations team led by Dr. Mila Pinchev; the investigators of the Australia New Zealand NRG Oncology group; members and participants in the Ontario Cancer Genetics Network; Leigha Senter, Kevin Sweet, Caroline Craven, Julia Cooper, and Michelle O'Conor; Yip Cheng Har, Nur Aishah Mohd Taib, Phuah Sze Yee, Norhashimah Hassan and all the research nurses, research assistants and doctors involved in the MyBrCa Study for assistance in patient recruitment, data collection and sample preparation, Philip Iau, Sng Jen-Hwei and Sharifah Nor Akmal for contributing samples from the Singapore

Breast Cancer Study and the HUKM-HKL Study respectively; the Meirav Comprehensive breast cancer center team at the Sheba Medical Center; Christina Selkirk; Cecilia Zvocec, Qun Niu; Joyce Seldon and Lorna Kwan; Dr. Robert Nussbaum, Beth Crawford, Kate Loranger, Julie Mak, Nicola Stewart, Robin Lee, Amie Blanco and Peggy Conrad and Salina Chan; Simon Gayther, Susan Ramus, Paul Pharoah, Carole Pye, Patricia Harrington and Eva Wozniak; Geoffrey Lindeman, Marion Harris, Martin Delatycki, Sarah Sawyer, Rebecca Driessen, and Ella Thompson for performing all DNA amplification.

The CIMBA data management and data analysis were supported by Cancer Research – UK grants C12292/A20861, C12292/A11174. ACA is a Cancer Research -UK Senior Cancer Research Fellow. GCT and ABS are NHMRC Research Fellows. Genotyping for the OncoArray was funded by the government of Canada through Genome Canada and the Canadian Institutes of Health Research (GPH-129344), the Ministère de l'Économie, de la Science et de l'Innovation du Québec through Génome Québec, the Quebec Breast Cancer Foundation for the PERSPECTIVE project, the US National Institutes of Health (NIH) (1 U19 CA 148065 for the Discovery, Biology and Risk of Inherited Variants in Breast Cancer (DRIVE) project and X01HG007492 to the Center for Inherited Disease Research (CIDR) under contract HHSN268201200008I), Cancer Research UK (C1287/A16563), the Odense University Hospital Research Foundation (Denmark), the National R&D Program for Cancer Control–Ministry of Health and Welfare (Republic of Korea) (1420190), the Italian Association for Cancer Research (AIRC; IG16933), the Breast Cancer Research Foundation, the National Health and Medical Research Council (Australia) and German Cancer Aid (110837).

BCFR-AU / BCFR-NC: UM1 CA164920 from the U.S. National Cancer Institute. The content of this manuscript does not necessarily reflect the views or policies of the National Cancer Institute or any of the collaborating centers in the Breast Cancer Family Registry (BCFR), nor does mention of trade names, commercial products, or organizations imply endorsement by the U.S. Government or the BCFR.

BCFR-ON: The Ontario Familial Breast Cancer Registry was supported by grant U01CA164920 from the U.S. National Cancer Institute of the National Institutes of Health. The content of this manuscript does not necessarily reflect the views or policies of the National Cancer Institute or any of the collaborating centers in the Breast Cancer Family Registry (BCFR), nor does mention of trade names, commercial products, or organizations imply endorsement by the U.S. Government or the BCFR.

COH: Research reported in this publication was supported by the National Cancer Institute of the National Institutes of Health under grant number R25CA112486, and RC4CA153828 (PI: J. Weitzel) from the National Cancer Institute and the Office of the Director, National Institutes of Health. The content is solely the responsibility of the authors and does not necessarily represent the official views of the National Institutes of Health.

DEMOKRITOS: European Union (European Social Fund – ESF) and Greek national funds through the Operational Program "Education and Lifelong Learning" of the National Strategic Reference Framework (NSRF) - Research Funding Program of the General Secretariat for Research & Technology: SYN11_10_19 NBCA. Investing in knowledge society through the European Social Fund.

EMBRACE: Cancer Research UK Grants C1287/A10118 and C1287/A11990. D. Gareth Evans and Fiona Lalloo are supported by an NIHR grant to the Biomedical Research Centre, Manchester. The Investigators at The Institute of Cancer Research and The Royal Marsden NHS Foundation Trust are supported by an NIHR grant to the Biomedical Research Centre at The Institute of Cancer Research and The Royal Marsden NHS Foundation Trust. Ros Eeles and Elizabeth Bancroft are supported by Cancer Research UK Grant C5047/A8385. Ros Eeles is also supported by NIHR support to the Biomedical Research Centre at The Institute of Cancer Research and The Royal Marsden NHS Foundation Trust.

GC-HBOC: German Cancer Aid (grant no 110837, Rita K. Schmutzler) and the European Regional Development Fund and Free State of Saxony, Germany (LIFE - Leipzig Research Centre for Civilization Diseases, project numbers 713-241202, 713-241202, 14505/2470, 14575/2470).

HEBCS: Helsinki University Hospital Research Fund, Academy of Finland (266528), the Finnish Cancer Society and the Sigrid Juselius Foundation.

HEBON: the Dutch Cancer Society grants NKI1998-1854, NKI2004-3088, NKI2007-3756, the Netherlands Organization of Scientific Research grant NWO 91109024, the Pink Ribbon grants 110005 and 2014-187.WO76, the BBMRI grant NWO 184.021.007/CP46 and the Transcan grant JTC 2012 Cancer 12-054. HEBON thanks the registration teams of Dutch Cancer Registry (IKNL; S. Siesling, J. Verloop) and the Dutch Pathology database (PALGA; L. Overbeek) for part of the data collection.

ICO: The authors would like to particularly acknowledge the support of the Asociación Española Contra el Cáncer (AECC), the Instituto de Salud Carlos III (organismo adscrito al Ministerio de Economía y Competitividad) and “Fondo Europeo de Desarrollo Regional (FEDER), una manera de hacer Europa” (PI10/01422, PI13/00285, PIE13/00022, PI15/00854, PI16/00563 and CIBERONC) and the Institut Català de la Salut and Autonomous Government of Catalonia (2009SGR290, 2014SGR338 and PERIS Project MedPerCan).

IHCC: PBZ_KBN_122/P05/2004.

IPOBCS: Liga Portuguesa Contra o Cancro.

kConFab: The National Breast Cancer Foundation, and previously by the National Health and Medical Research Council (NHMRC), the Queensland Cancer Fund, the Cancer Councils of New South Wales, Victoria, Tasmania and South Australia, and the Cancer Foundation of Western Australia. MAYO: NIH grants CA116167, CA192393 and CA176785, an NCI Specialized Program of Research Excellence (SPORE) in Breast Cancer (CA116201),and a grant from the Breast Cancer Research Foundation.

SWE-BRCA: the Swedish Cancer Society.

UPENN: Breast Cancer Research Foundation; Susan G. Komen Foundation for the cure, Basser Research Center for BRCA.

VFCTG: Victorian Cancer Agency, Cancer Australia, National Breast Cancer Foundation.

Consortia

kConFab Investigators

Amanda B. Spurdle[1], Georgia Chenevix-Trench[1], Jonathan Beesley[1], Xiao Qing Chen[1], Helene Holland[1], Heather Thorne[2], Stephen Fox[2], Eveline NiederMayr[2]

HEBON Investigators

Maartje J. Hooning[3], Matti A. Rookus[4], Frans B.L. Hogervorst[5], Flora van Leeuwen[5], Muriel A. Adank[5], Denise Jenner[5], Johanna M. Collée[6], Ans M.W. van den Ouweland[7], Ingrid A. Boere[8], Christi J. van Asperen[9], Peter Devilee[10, 11], Rob B. van der Luijt[12], T.C.T.E.F. van Cronenburg[13], M.R. Wevers[14], Arjen R. Mensenkamp[15], Margreet G.E.M. Ausems[12], Marco Koudijs[12], Theo A.M. van Os[16], Klaartje van Engelen[17],

Johan J.P. Gille[17], Encarna B. Gómez Garcia[18], Marinus J. Blok[19], Maaike de Boer[20], Lieke P.V. Berger[21], Annemieke H. van der Hout[22], Marian J.E. Mourits[21], Geertruida H. de Bock[21], Sabine Siesling[23], Janneke Verloop[23], Esther C. van den Broek[24]

SWE-BRCA Investigators

Ake Borg[25], Håkan Olsson[26], Helena Jernström[27], Karin Henriksson[28], Katja Harbst[27], Maria Soller[29], Ulf Kristoffersson[27], Anna Öfverholm[30], Margareta Nordling[30], Per Karlsson[31], Zakaria Einbeigi[31], Anna von Wachenfeldt[32], Annelie Liljegren[32], Annika Lindblom[33, 34], Brita Arver[32], Gisela Barbany Bustinza[34], Johanna Rantala[35], Beatrice Melin[36], Christina Edwinsdotter Ardnor[37], Monica Emanuelsson[36], Hans Ehrencrona[29], Maritta Hellström-Pigg[38], Richard Rosenquist[38], Marie Stenmark-Askmalm[39], Sigrun Liedgren[39]

[1] QIMR Berghofer Medical Research Institute, Department of Genetics and Computational Biology, Brisbane, Queensland, Australia.
[2] Peter MacCallum Cancer Center, Research Department, Melbourne, Victoria, Australia.
[3] Erasmus MC Cancer Institute, Department of Medical Oncology, Family Cancer Clinic, Rotterdam, The Netherlands.
[4] The Netherlands Cancer Institute, Department of Epidemiology, Amsterdam, The Netherlands.
[5] The Netherlands Cancer Institute - Antoni van Leeuwenhoek hospital, Family Cancer Clinic, Amsterdam, The Netherlands.
[6] Erasmus University Medical Center, Rotterdam, The Netherlands.
[7] Erasmus University Medical Center, Department of Clinical Genetics, Rotterdam, The Netherlands.
[8] Erasmus MC Cancer Institute, Department of Medical Oncology, Rotterdam, The Netherlands.
[9] Leiden University Medical Center, Department of Clinical Genetics, Leiden, The Netherlands.
[10] Leiden University Medical Center, Department of Pathology, Leiden, The Netherlands.
[11] Leiden University Medical Center, Department of Human Genetics, Leiden, The Netherlands.
[12] University Medical Center, Department of Medical Genetics, Utrecht, The Netherlands.
[13] Leiden University Medical Center, Leiden, The Netherlands.
[14] Radboud University Medical Center, Nijmegen, The Netherlands.
[15] Radboud University Medical Center, Department of Human Genetics, Nijmegen, The Netherlands.
[16] Amsterdam UMC, location AMC, Department of Clinical Genetics, Amsterdam, The Netherlands.
[17] VU University Medical Center, Department of Clinical Genetics, Amsterdam, The Netherlands.
[18] Maastricht University Medical Center, Department of Clinical Genetics and GROW, School for Oncology and Developmental Biology, Maastricht, The Netherlands.
[19] Maastricht University Medical Center, Department of Clinical Genetics, Maastricht, The Netherlands.
[20] Maastricht University Medical Center, Maastricht, The Netherlands.
[21] University Medical Center Groningen, University Groningen, Groningen, The Netherlands.
[22] University Medical Center Groningen, University Groningen, Department of Genetics, Groningen, The Netherlands.
[23] Netherlands Comprehensive Cancer Organisation (IKNL), Utrecht, The Netherlands.
[24] Erasmus University, The nationwide network and registry of histo- and cytopathology in The Netherlands (PALGA), Rotterdam, The Netherlands.
[25] Lund University and Skåne University Hospital, Department of Oncology, Lund, Sweden.
[26] Lund University, Department of Cancer Epidemiology, Clinical Sciences, Lund, Sweden.
[27] Lund University, LUCC - Lund University Cancer Centre, Lund, Sweden.
[28] Lund University Hospital, Oncologic Centre, Regional Tumour Registry, Lund, Sweden.
[29] Lund University Hospital, Department of Clinical Genetics, Lund, Sweden.
[30] Sahlgrenska University Hospital, Department of Clinical Genetics, Gothenburg, Sweden.
[31] Sahlgrenska University Hospital, Department of Oncology, Gothenburg, Sweden.
[32] Karolinska Institutet, Department of Oncology, Stockholm, Sweden.
[33] Karolinska Institutet, Department of Molecular Medicine and Surgery, Stockholm, Sweden.
[34] Karolinska University Hospital, Department of Clinical Genetics, Stockholm, Sweden.
[35] Karolinska Institutet, Clinical Genetics, Stockholm, Sweden.
[36] Umeå University, Department of Radiation Sciences, Oncology, Umeå, Sweden.
[37] Changhua Christian Hospital, Department of Surgery, Changhua, Taiwan.
[38] Uppsala University, Department of Immunology, Genetics and Pathology, Uppsala, Sweden.
[39] Linköping University, Division of Clinical Genetics, Department of Clinical and Experimental Medicine, Faculty of Health Sciences, Linköping, Sweden.

Supplementary Data 1. Identification of potential target genes based on variant position and functional annotations. (separate .xlsx file)

Supplementary Data 2. eQTL-analysis in GTEx and Westra et al. data. (separate .xlsx file)
