# Supplementary material for: Association of germline variation with the survival of women with BRCA1/2 pathogenic variants and breast cancer
Source: NPJ Breast Cancer. 2020 Sep 10;6:44. doi: 10.1038/s41523-020-00185-6 (PMC7483417; doi:10.1038/s41523-020-00185-6)
Supplement: Supplementary file 4 — Reporting summary checklist [file 41523_2020_185_MOESM4_ESM.pdf]

nature research

Corresponding author(s): Taru A. Muranen

Last updated by author(s): Dec 20, 2019

Reporting Summary

Nature Research wishes to improve the reproducibility of the work that we publish. This form provides structure for consistency and transparency in reporting. For further information on Nature Research policies, see Authors & Referees and the Editorial Policy Checklist.

Statistics

For all statistical analyses, confirm that the following items are present in the figure legend, table legend, main text, or Methods section.

n/a	Confirmed	
☐	☒	The exact sample size (n) for each experimental group/condition, given as a discrete number and unit of measurement
☐	☒	A statement on whether measurements were taken from distinct samples or whether the same sample was measured repeatedly
☐	☒	The statistical test(s) used AND whether they are one- or two-sided<br>*Only common tests should be described solely by name; describe more complex techniques in the Methods section.*
☐	☒	A description of all covariates tested
☐	☒	A description of any assumptions or corrections, such as tests of normality and adjustment for multiple comparisons
☐	☒	A full description of the statistical parameters including central tendency (e.g. means) or other basic estimates (e.g. regression coefficient) AND variation (e.g. standard deviation) or associated estimates of uncertainty (e.g. confidence intervals)
☐	☒	For null hypothesis testing, the test statistic (e.g. F, t, r) with confidence intervals, effect sizes, degrees of freedom and P value noted<br>*Give P values as exact values whenever suitable.*
☐	☒	For Bayesian analysis, information on the choice of priors and Markov chain Monte Carlo settings
☒	☐	For hierarchical and complex designs, identification of the appropriate level for tests and full reporting of outcomes
☐	☒	Estimates of effect sizes (e.g. Cohen's d, Pearson's r), indicating how they were calculated

Our web collection on statistics for biologists contains articles on many of the points above.

Software and code

Policy information about availability of computer code

Data collection	SNP calling was performed with Gentrain2 algorithm and imputation with SHAPEIT2 and IMPUTE version2 as described in Amos et al. 2017 Cancer Epidemiol. Biomarkers Prev. and in Michailidou et al. 2017 Nature.
Data analysis	Data was analyzed with R environment for statistical computing versions 3.5.1 and 3.6.0. R libraries included survival, MetaFor, and Gap.

For manuscripts utilizing custom algorithms or software that are central to the research but not yet described in published literature, software must be made available to editors/reviewers. We strongly encourage code deposition in a community repository (e.g. GitHub). See the Nature Research guidelines for submitting code & software for further information.

Data

Policy information about availability of data

All manuscripts must include a data availability statement. This statement should provide the following information, where applicable:

- Accession codes, unique identifiers, or web links for publicly available datasets
- A list of figures that have associated raw data
- A description of any restrictions on data availability

All summary results will be made available on the CIMBA website upon publication.

Field-specific reporting

Please select the one below that is the best fit for your research. If you are not sure, read the appropriate sections before making your selection.

☒ Life sciences ☐ Behavioural & social sciences ☐ Ecological, evolutionary & environmental sciences

For a reference copy of the document with all sections, see nature.com/documents/nr-reporting-summary-flat.pdf

Life sciences study design

All studies must disclose on these points even when the disclosure is negative.

Sample size	Sample size was determined by the number women with European ethnicity, pathogenic germline BRCA1/2 variants, and available genotype and follow-up data, from studies participating in the CIMBA consortium. Sample was as large as possible.
Data exclusions	CIMBA studies were not considered informative, if the number of cases under observation was less than 15 during the time that five deaths occurred. These studies were excluded.
Replication	The association of the candidate genes with patient survival was examined using the KM-plotter for breast cancer and published literature. Exact replication is not possible, since no other equally large collections of BRCA1/2 carriers exist.
Randomization	Randomization was not relevant in this time series analysis. We followed the survival of specific groups of breast cancer patients.
Blinding	The group allocation here was dead and alive at the end of follow-up. All patients were collected when alive and considered to be under observation only after recruitment to the study. Therefore the investigators recruiting the patients were not aware of the group allocation in advance. All patients received the best standard care for breast cancer.

Reporting for specific materials, systems and methods

We require information from authors about some types of materials, experimental systems and methods used in many studies. Here, indicate whether each material, system or method listed is relevant to your study. If you are not sure if a list item applies to your research, read the appropriate section before selecting a response.

Materials & experimental systems

n/a	Involved in the study	
☒	☐	Antibodies
☒	☐	Eukaryotic cell lines
☒	☐	Palaeontology
☒	☐	Animals and other organisms
☐	☒	Human research participants
☒	☐	Clinical data

Methods

n/a	Involved in the study	
☒	☐	ChIP-seq
☒	☐	Flow cytometry
☒	☐	MRI-based neuroimaging

Human research participants

Policy information about studies involving human research participants

Population characteristics	This study included women with European ethnicity, pathogenic germline variants in BRCA1/2 and diagnosis of invasive breast carcinoma.
Recruitment	Patients were recruited by studies participating in the CIMBA consortium from hospital-based series and familial cancer clinics.
Ethics oversight	All participating studies were approved by their appropriate ethics review boards.

Note that full information on the approval of the study protocol must also be provided in the manuscript.
